# Living on the Rocks: Genomic Analysis of Limestone Langurs Provides Novel Insights into the Adaptive Evolution in Extreme Karst Environments

**DOI:** 10.1093/gpbjnl/qzaf007

**Published:** 2025-02-11

**Authors:** Zhijin Liu, Xiongfei Zhang, Peipei Wang, Minheng Hong, Xiaochan Yan, Xiaoqiu Qi, Qian Zhao, Zhenghao Chen, Huajian Nie, Hui Li, Ziwen Li, Liye Zhang, Jiwei Qi, Chaolei He, Nguyen Van Truong, Minh D Le, Tilo Nadler, Hiroo Imai, Christian Roos, Ming Li

**Affiliations:** College of Life Sciences, Capital Normal University, Beijing 100048, China; CAS Key Laboratory of Animal Ecology and Conservation Biology, Institute of Zoology, Chinese Academy of Sciences, Beijing 100101, China; College of Life Sciences, Capital Normal University, Beijing 100048, China; College of Life Sciences, Capital Normal University, Beijing 100048, China; College of Life Sciences, Capital Normal University, Beijing 100048, China; Center for the Evolutionary Origins of Human Behavior, Kyoto University, Inuyama 484-8506, Japan; College of Life Sciences, Capital Normal University, Beijing 100048, China; College of Life Sciences, Capital Normal University, Beijing 100048, China; College of Life Sciences, Capital Normal University, Beijing 100048, China; College of Life Sciences, Capital Normal University, Beijing 100048, China; College of Life Sciences, Capital Normal University, Beijing 100048, China; College of Life Sciences, Capital Normal University, Beijing 100048, China; Primate Genetics Laboratory, German Primate Center, Leibniz Institute for Primate Research, 37077 Göttingen, Germany; CAS Key Laboratory of Animal Ecology and Conservation Biology, Institute of Zoology, Chinese Academy of Sciences, Beijing 100101, China; College of Life Sciences, Capital Normal University, Beijing 100048, China; Primate Genetics Laboratory, German Primate Center, Leibniz Institute for Primate Research, 37077 Göttingen, Germany; Institute for Biochemistry and Biology, University of Potsdam, 14476 Potsdam, Germany; Central Institute for Natural Resources and Environmental Studies, Vietnam National University, Hanoi 110403, Vietnam; Central Institute for Natural Resources and Environmental Studies, Vietnam National University, Hanoi 110403, Vietnam; Faculty of Environmental Sciences, University of Science, Vietnam National University, Hanoi 110403, Vietnam; Three Monkeys Wildlife Conservancy, Nho Quan 08400, Vietnam; Center for the Evolutionary Origins of Human Behavior, Kyoto University, Inuyama 484-8506, Japan; Primate Genetics Laboratory, German Primate Center, Leibniz Institute for Primate Research, 37077 Göttingen, Germany; Gene Bank of Primates, German Primate Center, Leibniz Institute for Primate Research, 37077 Göttingen, Germany; CAS Key Laboratory of Animal Ecology and Conservation Biology, Institute of Zoology, Chinese Academy of Sciences, Beijing 100101, China; Center for Excellence in Animal Evolution and Genetics, Chinese Academy of Sciences, Kunming 650223, China

**Keywords:** Non-human primate, Karst environment, Ion channel, DNA damage response, Camouflage

## Abstract

Understanding how organisms adapt to their environments is a central question in evolutionary biology. Limestone langurs are unique among primates, as they are exclusively found in karst limestone habitats and have evolved mechanisms to tolerate high levels of mineral ions, which are typically associated with metal toxicity affecting organs, cells, and genetic material. We generated a high-quality reference genome (Tfra_5.0) for the limestone langur (*Trachypithecus francoisi*), along with genome resequencing data for 48 langurs representing 15 *Trachypithecus* species. Genes encoding ion channels (*e.g.*, Na^+^, K^+^, and Ca^2+^) exhibited significantly accelerated evolution in limestone langurs. Limestone langur-specific mutations in Na^+^ and Ca^2+^ channels were experimentally confirmed to modify inward ion currents *in vitro*. Unexpectedly, scans for positive selection also identified genes involved in DNA damage response/repair pathways, a previously unknown adaptation. This finding highlights an evolutionary adaptation in limestone langurs that mitigates the increased risk of DNA damage posed by elevated metal ion concentrations. Notably, a limestone langur-specific mutation (E94D) of the melanocortin 1 receptor (MC1R) was associated with increased basal cyclic adenosine monophosphate (cAMP) production, contributing to the species’ darker coat color, which likely serves as camouflage on limestone rocks. Our findings reveal novel adaptive evolutionary mechanisms of limestone langurs and offer broader insights into organismal adaptation to extreme environments, with potential implications for understanding human health, biological evolution, and biodiversity conservation.

## Graphical abstract

**Graphical abstract:**
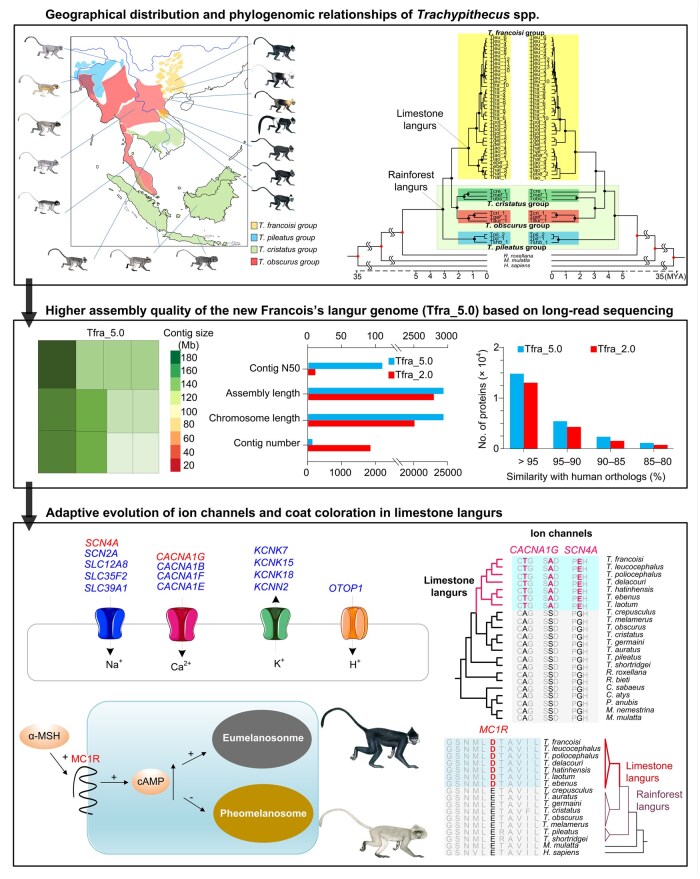

## Introduction

Organisms inhabiting extreme environments serve as unique models for exploring how they have adapted and how they cope with various extrinsic factors and challenges. With over 500 extant species, the order Primates is one of the most speciose mammalian groups, exhibiting extensive behavioral, morphological, and physiological diversity across a wide range of habitats. Recent advances in genome sequencing and assembly technologies have significantly advanced genomics research, offering deeper insights into the biology and evolution of organisms. For primates, genome assemblies and resequencing data are now accessible for multiple species, providing crucial insights into the biology and evolution of both human and non-human primates (NHPs) [1,2]. A recent phylogenomic analysis of 50 primate reference genomes identified thousands of positively selected genes (PSGs) involved in digestive, skeletal, and nervous systems, which have contributed to evolutionary innovations and adaptations in various primate lineages [2]. While resequencing data have been published for nearly half of all primate species [1], high-quality genome references are available for only ∼ 10% of species [2]. Thus, expanding high-quality genomic resources to additional species will offer further insights into primate evolution, benefiting both the species under investigation and the primates as a whole. In particular, identifying PSGs and rapidly evolving genes (REGs) within specific lineages, as well as genes linked to distinct traits, will yield further insights into the mechanisms crucial for adaptation, organismal health and survival, and the biology and biomedicine of humans.

Karst limestone landscapes cover more than 10% of global continental land areas, and Southeast Asia’s karst region, spanning approximately 500,000 km^2^, is one of the largest in the world [3]. Exposed bare rocks and hills are common features of limestone regions. Moreover, karst limestone environments are characterized by elevated calcium (Ca^2+^) levels, reduced water retention capacity, and nutrient deficiencies, except for certain metal ions such as Ca^2+^ and magnesium (Mg^2+^), compared to non-karst soils [4]. Adaptations to the high Ca^2+^ concentration in karst environments have been reported in various plants and animals, primarily focusing on the evolution of calcium ion channels [5–8]. However, comprehensive genomic adaptations to other forms of metal toxicity in karst environments have been less thoroughly explored in organisms.

**Figure 1:**
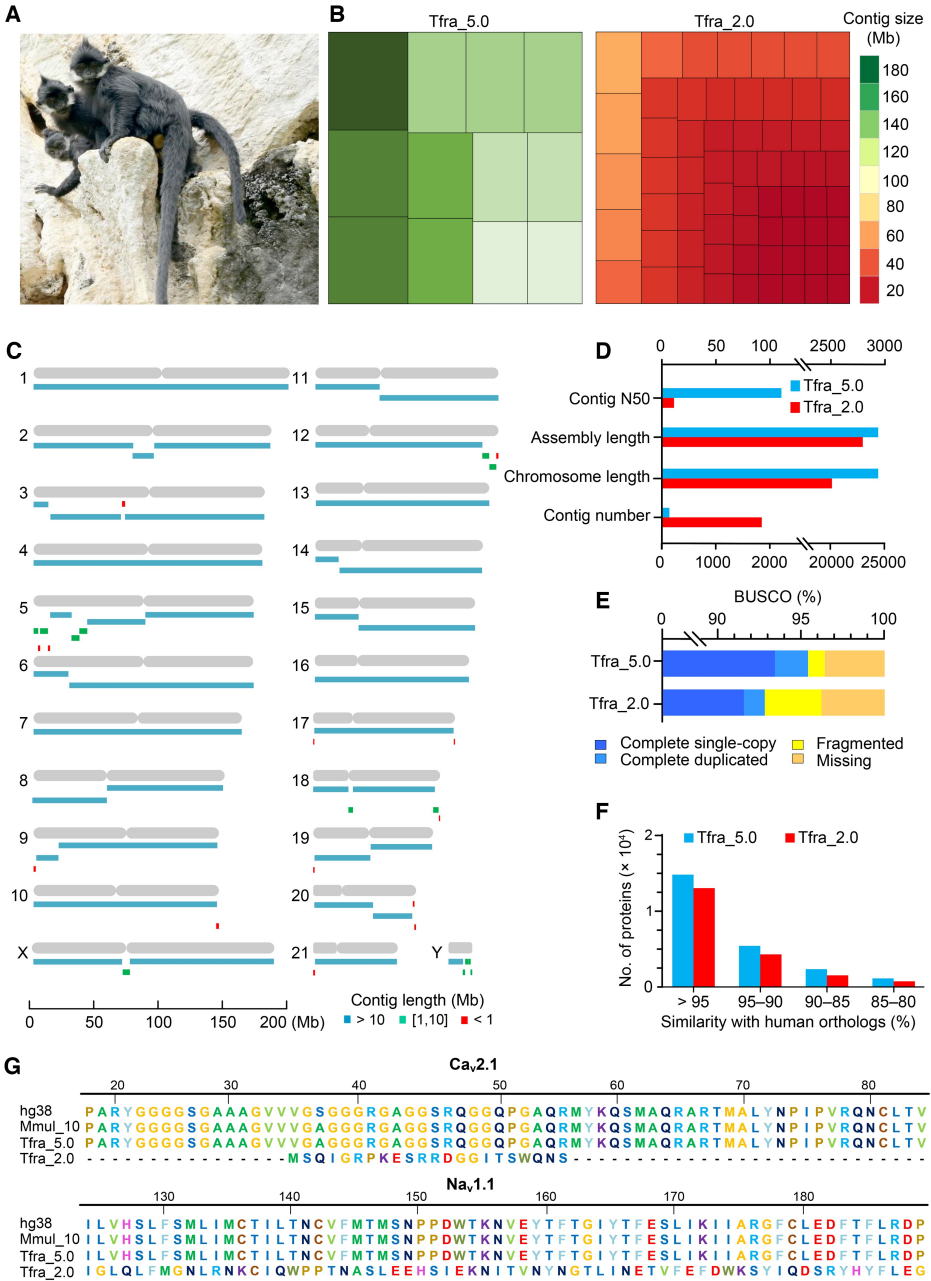
Assembly quality of the newly sequenced Francois’s langur genome (Tfra_5.0) based on long-read sequencing **A**. Photograph of limestone langurs (*Trachypithecus francoisi*, photographed by Jiaxin Zhao). **B**. Treemaps illustrating genome fragmentation disparities between Tfra_5.0 and Tfra_2.0. The rectangular units depict the largest contigs constituting of ∼ 1.5 Gb (∼ 50%) of Tfra_5.0 and Tfra_2.0, respectively. **C**. Chromosomal pattern of Tfra_5.0 with contigs. The assembled contigs encompass segments > 10 Mb (blue), 1−10 Mb (green), and < 1 Mb (red). **D**. Comparison of contig N50, assembly length, chromosome length, and contig number between Tfra_5.0 and Tfra_2.0. **E**. Comparison of BUSCO results between Tfra_5.0 and Tfra_2.0. **F**. Similarity comparison of proteins extracted from Tfra_5.0 and Tfra_2.0 to human (hg38) orthologs. **G**. Completeness comparison of ion channel proteins Ca_v_2.1 and Na_v_1.1 in human (hg38), rhesus macaque (Mmul_10), and *T. francoisi* (Tfra_5.0, and Tfra_2.0). BUSCO, Benchmarking Universal Single-Copy Orthologs.

The Southeast Asian karst region encompasses a range of landforms in various humid, subhumid, tropical, and subtropical climatic and geographical settings [3,4]. Limestone langurs (Primates, Cercopithecidae) are unique among primates, as they are endemic to the limestone habitats of Southeast Asia. They belong to the genus *Trachypithecus,* which comprises 22 species divided into four groups [9]. Three of these groups (the *Trachypithecus cristatus*, *Trachypithecus pileatus*, and *Trachypithecus obscurus* groups) primarily inhabit rainforest environments, whereas the fourth group, the *Trachypithecus francoisi* group (limestone langurs), is found in limestone karst habitats (**Figure 1**A) [9]. The primary environmental stressor for limestone langurs is the high ion content found in carbonate rocks, soil, water, and karst plants that serve as their food sources [10,11]. Molecular adaptations that protect cells from damage caused by high concentrations of metal ions may be a key evolutionary trait in limestone langurs, making them an ideal and unique system for studying adaptation to such environmental challenges. In addition, significant morphological differences exist between limestone and rainforest langurs. Rainforest langurs typically exhibit predominantly gray or brown dorsal fur and lighter ventral surfaces [9]. In contrast, limestone langurs have primarily black fur, which is thought to serve as camouflage by mimicking the shadow of rocks on bare karst surfaces, helping them avoid predators [12]. When light falls from above, the back fur of limestone langurs merges with shadows to obscure their three-dimensional form, creating a two-dimensional appearance that makes them harder to detect as living animals [12]. However, knowledge about the genetic mechanisms underlying this adaptive phenotypic trait remains limited due to the lack of high-quality genomic resources.

Although a draft genome assembly (Tfra_2.0) of a limestone langur (*T. francoisi*) exists, its quality is suboptimal, containing gaps, sequencing errors, misassemblies, and poor annotation [6]. Using Tfra_2.0, a limestone langur-specific amino acid (AA) mutation in calcium channel Ca_v_1.2 (encoded by *CACNA1C*) was identified, which reduces calcium influx into cells and is thought to represent an adaptation to the karst environment [6]. However, the full breadth of adaptive evolutionary signals in other ion channels (*e.g.,* Na^+^, K^+^, and Ca^2+^) and pathways responding to high ion intake and metal toxicity can only be explored once a more complete genome assembly becomes available. Ion channels are proteins that selectively permit ions to diffuse across cell membranes, generating membrane potential that is critical for essential physiological processes [13]. Mutations in ion channels can result in a variety of cardiac diseases, neurological disorders, and other conditions collectively known as “channelopathies” in humans [13]. It is hypothesized that ion channels of limestone langurs have undergone adaptive evolution, potentially providing insights into how AA mutations affect ion channel function and the mechanisms underlying channelopathies. However, knowledge of adaptive evolution in the ion channels of limestone langurs remains limited, and further investigation is hindered by the lack of high-quality genomic resources.

A fundamental principle in genomics asserts that higher-quality genome assemblies enable more accurate annotations and facilitate precise genetic and evolutionary interpretations [14]. Here, we present a novel *de novo* assembly of the Francois’s langur (*T. francoisi*) (Figure 1A) genome (Tfra_5.0) using advanced PacBio high-fidelity (HiFi) and high-throughput chromosome conformation capture (Hi-C) sequencing technologies. Tfra_5.0 features five fully assembled chromosomes, a sixfold increase in contig N50 to 108.99 Mb compared to Tfra_2.0, and significantly improved annotation. Additionally, we generated whole-genome resequencing data (∼ 30× coverage) for 48 langur individuals from 15 species, representing all four species groups within the genus *Trachypithecus*. In this study, we aimed to address three key questions. (1) Is the adaptive evolution of ion channels in limestone langurs widespread, or is their adaptation to high metal ion intake limited to a few calcium channel AA mutations? (2) To what extent have the ion channel changes contributed to the adaptive evolution of limestone langurs, and are other pathways also involved in their response to metal toxicity? (3) Regarding morphological adaptation, what genetic mechanisms underlie the coat color difference between limestone and rainforest langurs? Overall, we performed a comprehensive investigation of genome evolution underlying adaptive traits in limestone langurs, uncovering novel mechanisms of adaptation to high metal ion intake, DNA damage, and coat coloration. These findings provide valuable insights into vertebrate biology, human health, and biological conservation.

## Results

### HiFi sequencing, genome assembly, annotation, and genome resequencing

A total of 8,787,764 filtered HiFi reads (159,146,517,525 bp; N50 = 18.72 kb; maximum length = 68.03 kb) were obtained. For genome scaffolding and evaluation, cleaned Hi-C reads (177.73 Gb) were retrieved from a previous study [6]. The Tfra_5.0 genome assembly is more complete, with fewer gaps and greater accuracy than Tfra_2.0 (Figure 1B–F). The 2.96-Gb genome assembly was accurately clustered into 21 pseudo-autosomes, one pseudo-X-chromosome, and one pseudo-Y-chromosome, representing ∼ 99.70% of the predicted genome size (Figure 1C, Figure S1; Table S1). In contrast, Tfra_2.0 assembled only 2.61 Gb (∼ 87.87% of the genome size) to chromosomal level. Tfra_5.0 exhibits 98.02% and 97.15% alignment with the *Macaca mulatta* (Mmul_10) and *Homo sapiens* (GRCh38.p14) genomes, respectively. We observed chromosomal rearrangements between *T. francoisi* and *M. mulatta*. For example, chromosome 10 of *M. mulatta* aligns with chromosomes 15 and 18 of *T. francoisi*, while chromosome 21 of *T. francoisi* encompasses segments from chromosomes 19 and 1 of *M. mulatta* (Figure S2).

Tfra_5.0 shows significantly less fragmentation and improved sequence contiguity compared to Tfra_2.0 (65 *vs*. 1740 contigs, a 27-fold decrease in number) (Figure 1D). The contig N50 length of Tfra_5.0 is 108.99 Mb, a sixfold improvement over the Tfra_2.0 contig N50 (16.32 Mb) (Figure 1D). The longest contig of Tfra_5.0 is 199.53 Mb, with a GC content of 41.38%. Interspersed repeats account for ∼ 50.80% of the Tfra_5.0 assembly (Table S2). Gene completion evaluation using Benchmarking Universal Single-Copy Orthologs (BUSCO; v5.4.2) [15] suggests that Tfra_5.0 contains 95.40% complete mammalian universal single-copy orthologs, higher than Tfra_2.0 (92.86%) (Figure 1E). A total of 24,635 protein-coding genes (19,856 with informative names) were predicted in Tfra_5.0, compared to 21,622 protein-coding genes (16,841 genes with informative names) in Tfra_2.0. Proteins extracted from Tfra_5.0 showed higher similarity to human (hg38) orthologs than Tfra_2.0 (Figure 1F). Notably, gaps and misassemblies in ion channel proteins Ca_v_2.1 and Na_v_1.1 present in Tfra_2.0 were corrected in Tfra_5.0 (Figure 1G).

**Figure 2:**
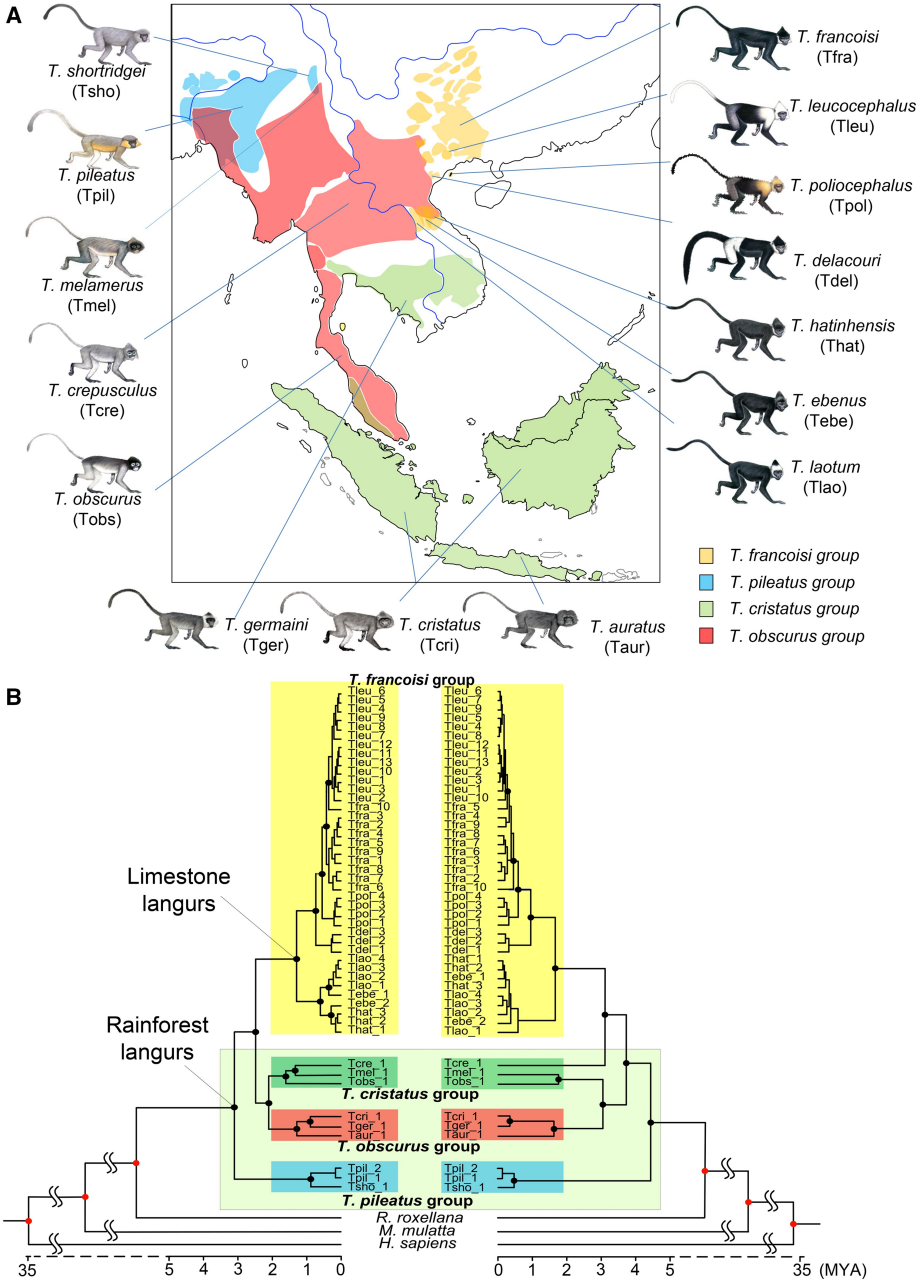
Geographical distribution and phylogenomic relationships of *Trachypithecus* spp. **A**. Distribution of the four species groups and approximate range of the 15 species investigated. **B**. Phylogeny and divergence time of *Trachypithecus* spp. inferred from autosomal SNPs (left) and mt-genomes (right). Branches and labels are color-coded to represent the four species groups. Nodes with black dots indicate ultrafast bootstrap values > 99%. MYA, million years ago; mt-genome, mitochondrial genome; SNP, single nucleotide polymorphism.

Furthermore, we collected and sequenced 48 individuals (25 individuals were newly sequenced) representing 15 *Trachypithecus* species, covering all four species groups: Francois’s langur (*T. francoisi*, 10 individuals), Cat Ba langur (*Trachypithecus poliocephalus*, 4 individuals), White-headed langur (*Trachypithecus leucocephalus*, 13 individuals), Delacour’s langur (*Trachypithecus delacouri*, 3 individuals), Laotian langur (*Trachypithecus laotum*, 4 individuals), Hatinh langur (*Trachypithecus hatinhensis*, 3 individuals), and Black langur (*Trachypithecus ebenus*, 2 individuals), representing all 7 species of the limestone langur group; Silvered langur (*T. cristatus*, 1 individual), East Javan langur (*Trachypithecus auratus*, 1 individual), and Germain’s langur (*Trachypithecus germaini*, 1 individual), as representatives of the *T. cristatus* group; Shan State langur (*Trachypithecus melamerus*, 1 individual), Indochinese gray langur (*Trachypithecus crepusculus*, 1 individual), and Dusky langur (*T. obscurus*, 1 individual), as representatives of the *T. obscurus* group; and Capped langur (*T. pileatus*, 2 individuals) and Shortridge’s langur (*Trachypithecus shortridgei*, 1 individual), as representatives of the *T. pileatus* group (**Figure 2**A; Table S3). High-depth whole-genome resequencing (30.06× ± 4.51×) was performed, and genome coverage on the Tfra_5.0 assembly ranged from 93.70% to 99.63% (98.26% ± 0.01%) (Table S3). We have assessed the level of DNA damage by mapDamage2.0 [16]. The C→T misconception at the 1st bp of the 5′-end of sequencing reads ranged from 0.0007–0.006 and the G→A misconception at the 1st bp of the 3′-end of sequencing reads ranged from 0.002–0.006 (Figure S3). This indicates that the degree of DNA damage is acceptable and suitable for genomic analysis using standard methods [17]. A total of 72,293,566 high-quality single nucleotide polymorphisms (SNPs) were obtained through variant calling and quality-filtering. Among these, 69,505,136 SNPs were located in autosomes and used for subsequent phylogenomic and comparative genomic analyses.

### Identification of 11,529 one-to-one orthologous genes among *T. francoisi* and nine primates

For comparative analysis, we examined the orthologous gene relationships among ten primates: *T. francoisi*, *T. melamerus*, *Rhinopithecus roxellana*, *Rhinopithecus bieti*, *Macaca fascicularis*, *M. mulatta*, *Papio anubis*, *Cercocebus atys*, *Pan troglodytes*, and *H*. *sapiens* through OrthoMCL [18]. In total, 17,325 gene families were clustered across these species, and 11,529 one-to-one orthologous genes were identified (Figure S4). Thirteen gene families expanded in *T. francoisi* after the divergence from *T. melamerus* (Figure S5A), and these genes were involved in Kyoto Encyclopedia of Genes and Genomes (KEGG) pathways including “RNA transport”, “mineral absorption”, “longevity regulating pathway”, and “GnRH signaling pathway” [false discovery rate (FDR) < 0.05, Fisher’s exact test] (Figure S5B).

### Limestone langurs diverged from rainforest langurs 2.44–3.04 million years ago

Maximum likelihood (ML) trees based on SNPs of autosomal protein coding sequences (CDSs) and mitochondrial genomes (mt-genomes) were reconstructed, respectively, incorporating 48 langurs of 15 *Trachypithecus* species and using other primates as outgroups (Figure 2B). According to the autosomal SNP tree, all four species groups form monophyletic clades (Figure S6A). The *T. pileatus* group diverged first, approximately 3.15 million years ago (MYA) [95% highest posterior density (HPD): 2.25–4.15 MYA], followed by the divergence of the *T. francoisi* group (limestone langurs) from the *T. cristatus* and *T. obscurus* groups 2.44 MYA (95% HPD: 1.86–3.42 MYA). The mt-genome tree topology is generally consistent with the autosomal phylogeny, but differs in the placement of *T. crepusculus* (Figure 2B, Figure S6B). The divergence between limestone langurs and rainforest langurs was estimated at 3.04 MYA (95% HPD: 2.49–3.55 MYA) based on the mt-genome data (Figure 2B, Figure S6B). The phylogenetic relationships based on X and Y chromosomal CDSs among *Trachypithecus* spp. are generally consistent with the autosomal phylogeny (Figure S7).

### PSGs and REGs were overrepresented in the Gene Ontology terms related to cation transmembrane transport and DNA damage response

**Figure 3:**
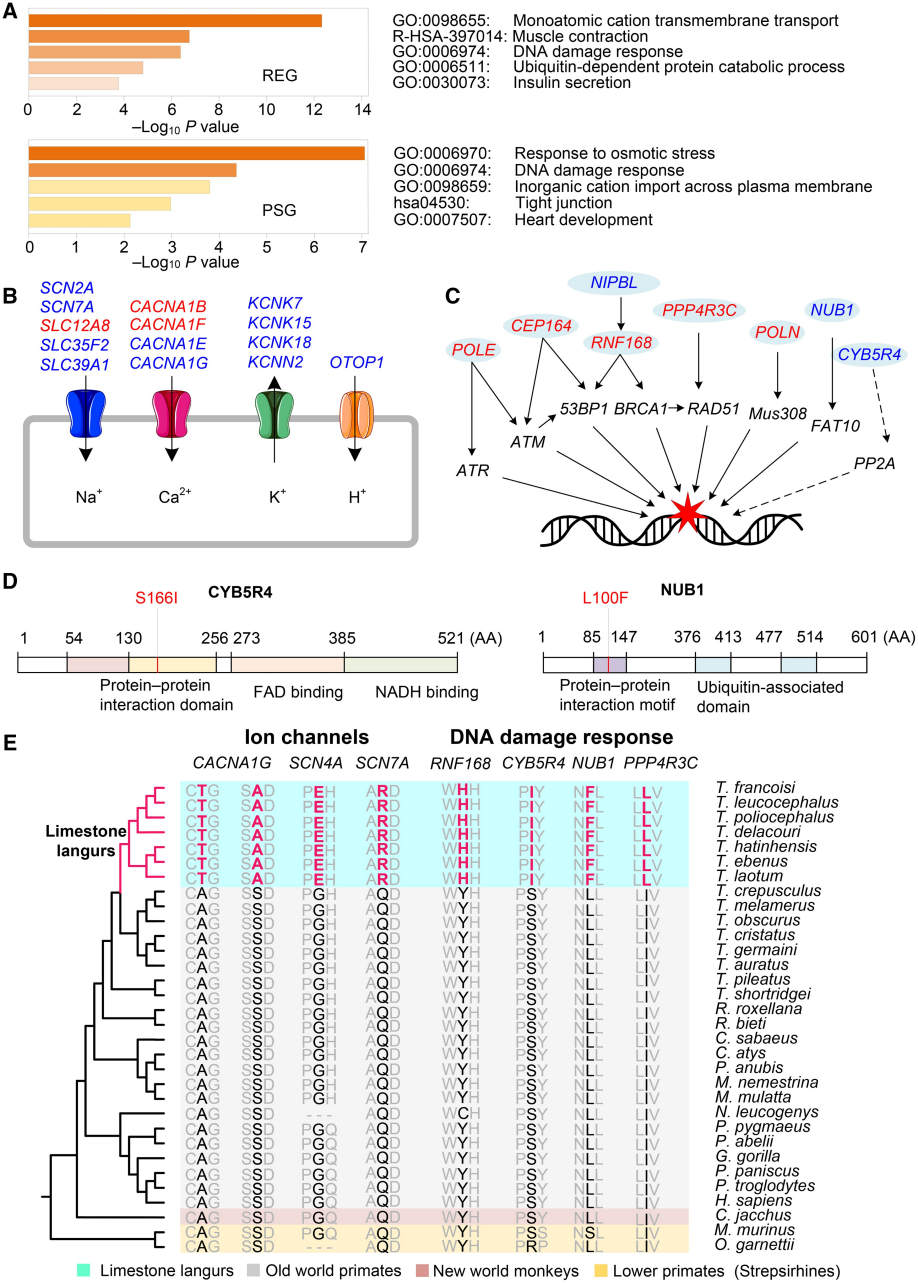
Adaptive evolution of ion channels and DNA damage response in limestone langurs **A**. GO enrichment analyses of REGs and PSGs in the *T. francoisi* lineage. **B**. REGs (blue) and PSGs (red) related to ion channels. **C**. REGs and PSGs involved in DNA damage response pathways. **D**. Limestone langur-specific AA mutations in the protein–protein interaction domains of CYB5R4 and NUB1. **E**. Multiple sequence alignment showing limestone langur-specific AA mutations in selected REGs and PSGs. AA, amino acid; PSG, positively selected gene; REG, rapidly evolving gene.

Using branch and branch-site models in codeML embedded in Phylogenetic Analysis by Maximum Likelihood (PAML; v4) [19], we identified 58 REGs (Chi-square test, *P* < 0.05) (Table S4) and 37 PSGs (Chi-square test, *P* < 0.05) (Table S5) in the *T. francoisi* lineage, compared to nine primate species used as background branches (*T. melamerus*, *R. roxellana*, *R. bieti*, *M. fascicularis*, *M. mulatta*, *P. anubis*, *C. atys*, *P. troglodytes*, and *H. sapiens*). REGs were overrepresented in the Gene Ontology (GO) term “monoatomic cation transmembrane transport” (GO: 0098655) with 18 genes (*ANXA6*, *CACNA1F*, *CACNA1G*, *CALHM6*, *COX15*, *KCNK7*, *KCNK18*, *KCNN2*, *KLF15*, *SCN2A*, *SCN7A*, *SLC12A8*, *SLC39A1*, *OTOP1*, *PDX1, PKD1L2*, *PIK3CG*, and *PSMD3*) (Fisher’s exact test, *P* < 1 × 10^−13^) (**Figure 3**A and B). They were also enriched in the GO term “muscle contraction” (R-HSA-397014) with 8 genes (*ANXA6*, *CACNA1G*, *KCNK7*, *KCNK18*, *KCNN2*, *SCN2A*, *SCN7A*, and *SLC12A8*) (Fisher’s exact test, *P* < 1 × 10^−6^) (Figure 3A), and the GO term “DNA damage response” (GO:0006974) containing 12 genes (*ASCC2*, *ASCC3*, *DONSON*, *GRWD1*, *NIPBL*, *NSMCE4A*, *POLN*, *PPP4R3C*, *REV3L*, *RIF1*, *RNF168*, and *SHPRH*) (Fisher’s exact test, *P* < 1 × 10^−6^) (Figure 3A and C). One notable REG identified in *T. francoisi*, *DMGDH*, is linked to human blood selenium concentration and selenium metabolism [20], a metal also commonly found in karst environments [21,22].

PSGs in *T. francoisi* showed enrichment in the GO terms “response to osmotic stress” (GO:0006970) with 5 genes (*MYLK*, *RCSD1*, *TH*, *TSC22D2*, and *WNK1*) (Fisher’s exact test, *P* < 1 × 10^−7^) (Figure 3A), “DNA damage response” (GO:0006974) with 9 genes (*AZI2*, *CEP164*, *DONSON*, *DOT1L*, *POLE*, *POLN*, *PPP4R3C*, *RNF168*, and *STAG2*) (Fisher’s exact test, *P* < 0.0001) (Figure 3A and C), and “inorganic cation import across plasma membrane” (GO:0098659) with 6 genes (*CACNA1B*, *CACNA1F*, *MYLK*, *NDUFB7*, *SLC12A8*, and *WNK1*) (Fisher’s exact test, *P* < 0.001) (Figure 3A and B). PSGs enriched in the GO term “response to DNA damage” [23] included 5 genes (*DOT1L*, *PPP4R3C*, *RNF168*, *STAG2*, and *WNK1*) (Fisher’s exact test, *P* < 0.001) (Figure 3C). Several limestone langur-specific AA mutations in REGs and PSGs are shown in Figure 3D and E.

### Molecular evolution of ion channels in *Trachypithecus* spp.

**Figure 4:**
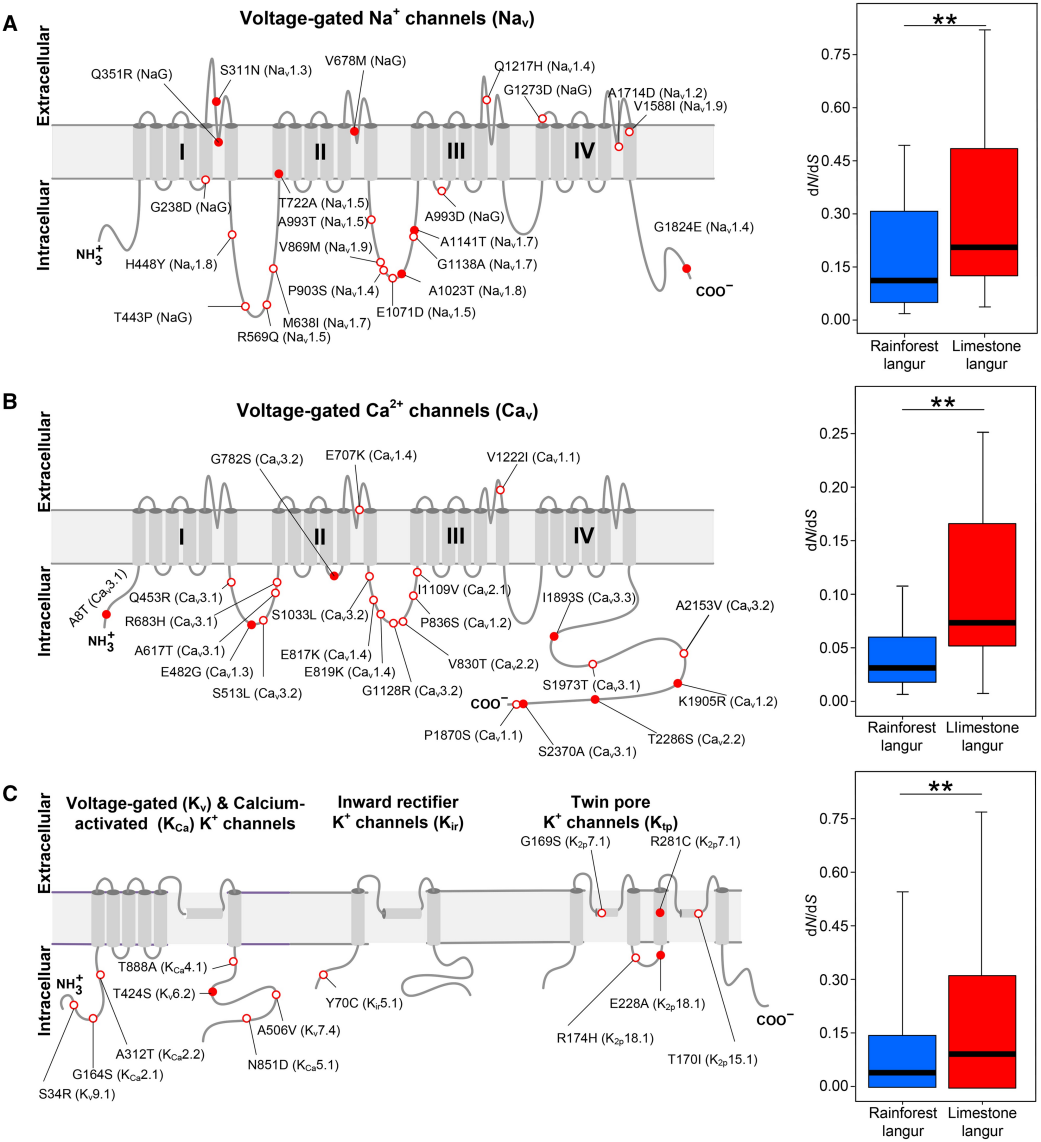
Molecular evolution of ion channels in limestone and rainforest langurs **A**. Left: structure and AA substitutions in voltage-gated sodium channels. Right: boxplot showing the d*N*/d*S* ratios of 10 sodium channel genes in limestone langurs compared to rainforest langurs (Wilcoxon two-sided test; **, *P* < 0.01). **B**. Left: structure and AA substitutions in voltage-gated calcium channels. Right: boxplot showing the d*N*/d*S* ratios of 10 calcium channel genes in limestone langurs compared to rainforest langurs (Wilcoxon two-sided test; **, *P* < 0.01). **C**. Left: structure and AA substitutions in potassium channels. Right: boxplot showing the d*N*/d*S* ratios of 80 potassium channel genes in limestone langurs compared to rainforest langurs (Wilcoxon two-sided test; **, *P* < 0.01). In (A–C), red filled and open circles represent substitutions found in all and some limestone langur species, respectively.

The annotation of 10 sodium channel genes (*SCN1A*–*SCN5A* and *SCN7A*–*SCN11A*) and 10 calcium channel genes (*CACNA1A*–*CACNA1I* and *CACNA1S*) in Tfra_5.0 is more complete and accurate than that in Tfra_2.0 (Figure S8). To fully investigate the molecular evolution of cation transmembrane channels in limestone langurs, orthologous genes encoding 10 Na_v_, 10 Ca_v_, and 80 potassium (K_v_, K_ir_, K_tp_, and K_Ca_) channels were retrieved from limestone and rainforest langurs (**Figure 4**A–C). Sixty limestone langur-specific AA substitutions were detected in these channel genes, with 18 fixed across all limestone langurs (Figure 4A–C; Tables S6 and S7). Using the branch model in PAML (v4) [19], we found that 10 calcium and 10 sodium channel proteins exhibited significantly elevated evolutionary rates (d*N*/d*S* ratios) in limestone langurs compared to rainforest langurs (Wilcoxon two-sided test, *P* < 0.01) (Figure 4A and B), as did the 80 potassium channels (Wilcoxon two-sided test, *P* < 0.01) (Figure 4C). Additionally, *CACNA1B*, *CACNA1C*, *CACNA1G*, *SCN2A*, *SCN3A*, *KCNC3*, *KCNK2*, *KCNK7*, *KCNN2*, and *KCNQ4* were identified as REGs.

**Figure 5:**
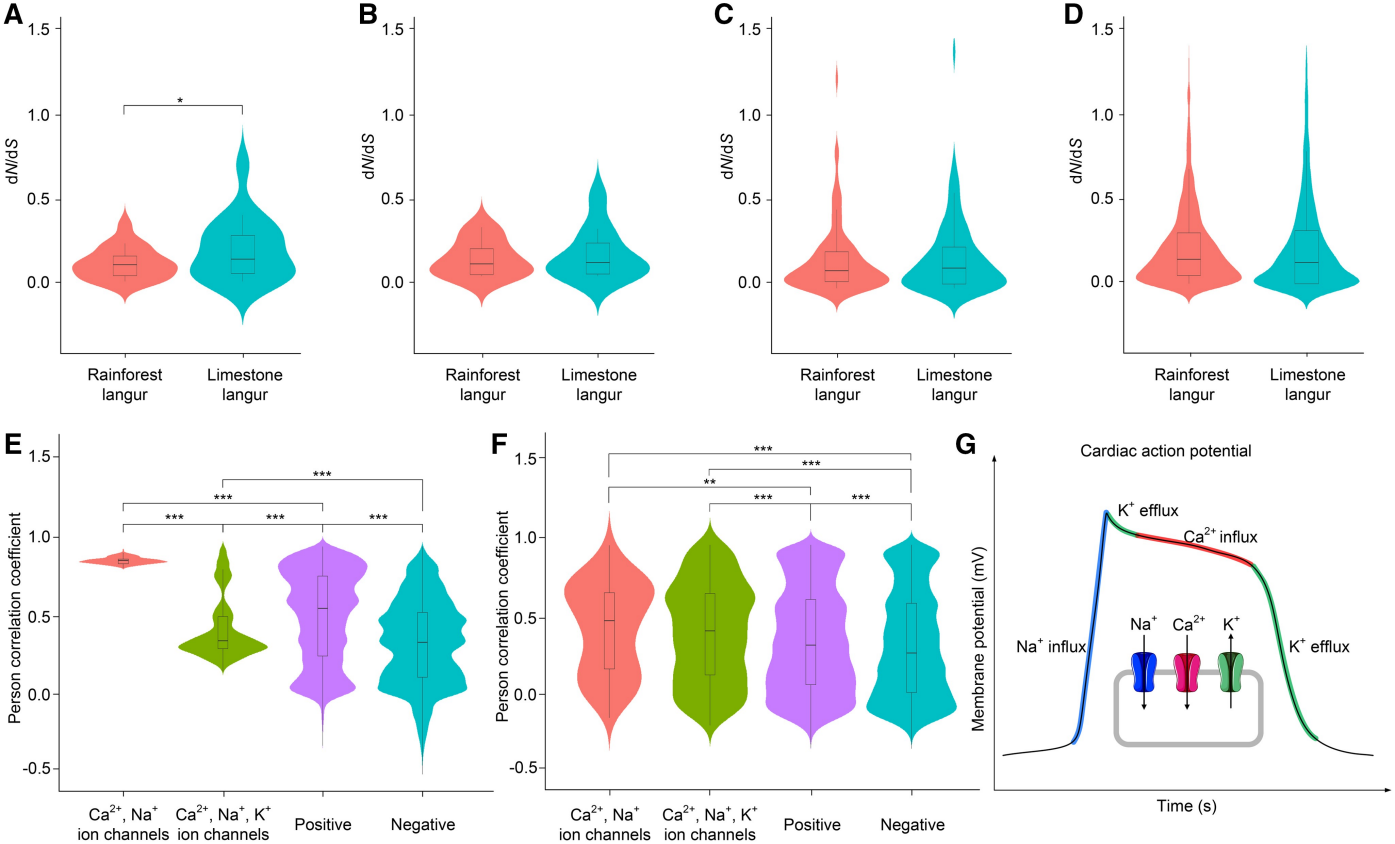
Molecular evolution of genes encoding ion channels in *Trachypithecus* spp. **A**.–**D**. d*N*/d*S* ratios and density distributions for 25 genes encoding the auxiliary subunits of sodium and calcium channels (A) (Wilcoxon two-sided test; *, *P* < 0.05), 16 genes encoding the auxiliary subunits of potassium channels (B) (Wilcoxon two-sided test; *P* > 0.05), 112 genes encoding other ion channels (C) (Student’s two-sided *t*-test; *P* > 0.05), and 1825 genes involved in the ion channel signaling pathways (D) (Student’s two-sided *t*-test; *P* > 0.05) in limestone and rainforest langurs. **E**. Comparison of coevolving lineages of ion channel proteins with positive and negative controls predicted by PrePhyloPro (Student’s two-sided *t*-test; ***, *P* < 0.001). **F**. Comparison of coevolving lineages of ion channel proteins with positive and negative controls predicted by MirrorTree (Student’s two-sided *t*-test; **, *P* < 0.01; ***, *P* < 0.001). **G**. Physiological changes of cardiac cell membranes mediated by ion channels.

We further calculated the d*N*/d*S* ratios for 25 genes encoding the auxiliary subunits of sodium and calcium channels and observed significantly higher d*N*/d*S* ratios in limestone langurs compared to rainforest langurs (Wilcoxon two-sided test, *P* < 0.05) (**Figure 5**A). Although the mean d*N*/d*S* ratio of 16 potassium channel auxiliary subunits was higher in limestone langurs (mean d*N*/d*S* = 0.133) than that in rainforest langurs (mean d*N*/d*S* = 0.108), the difference was not statistically significant (Wilcoxon two-sided test, *P* > 0.05) (Figure 5B). Furthermore, we retrieved orthologous sequences of 112 genes encoding cholinergic receptors, chloride channels, dopamine receptors, serotonin receptors, and ionotropic glutamate receptors [13] from both limestone and rainforest langur groups. While the mean d*N*/d*S* ratio for these genes was higher in limestone langurs than that in rainforest langurs, the difference was not statistically significant (Student’s two-sided *t*-test, *P* > 0.05) (Figure 5C). We further calculated the d*N*/d*S* ratios of 1825 genes involved in the ion channel signaling pathways as well as the density distribution of the d*N*/d*S* ratios in limestone and rainforest langurs, and found no statistically significant differences (Student’s two-sided *t*-test, *P* > 0.05) (Figure 5D).

### Higher coevolving levels of ion channels

Coevolution commonly occurs among proteins that are involved in pathway networks [24]. Coevolving lineages among the aforementioned ion channel proteins were predicted using PrePhyloPro online [25] based on the phylogenetic profiling of the cooccurrence across 972 eukaryotic and prokaryotic organisms, and were demonstrated by the Pearson correlation coefficients of protein pairs. The coevolving lineages among the 10 Na^+^ channels and 10 Ca^2+^ channels were significantly higher than the positive and negative datasets in PrePhyloPro (Student’s two-sided *t*-test, *P* < 0.001) (Figure 5E). We also employed MirrorTree to calculate the Pearson correlation coefficients between proteins based on the similarity of phylogenetic trees [24]. Protein orthologs from *T. francoisi* and other 12 mammals (*Sorex araneus*, *Myotis brandtii*, *Felis catus*, *H. sapiens*, *Gorilla gorilla*, *Bos taurus*, *M. mulatta*, *Canis familiaris*, *Sus scrofa*, *Mus musculus*, *Equus caballus*, and *Loxodonta africana*) were retrieved from OrthoMaM (v10c) [26]. Using the human protein binary interaction interactome as a reference [27], we found that the Pearson correlation coefficients among the Na^+^, K^+^, and Ca^2+^ channels were also significantly higher than those in control groups (Student’s two-sided *t*-test, *P* < 0.01) (Figure 5F). It implies that the coevolving levels among ion channels are higher than the average coevolving level among known coevolving proteins. These results suggest the evolutionary integrity of ion channels, because many fundamental physiological and cellular functions, such as myocardial contraction, are performed through the integrated dynamics of Na^+^, K^+^, and Ca^2+^ currents (Figure 5G).

### Limestone langur-specific AA substitutions decreased inward ion currents

**Figure 6:**
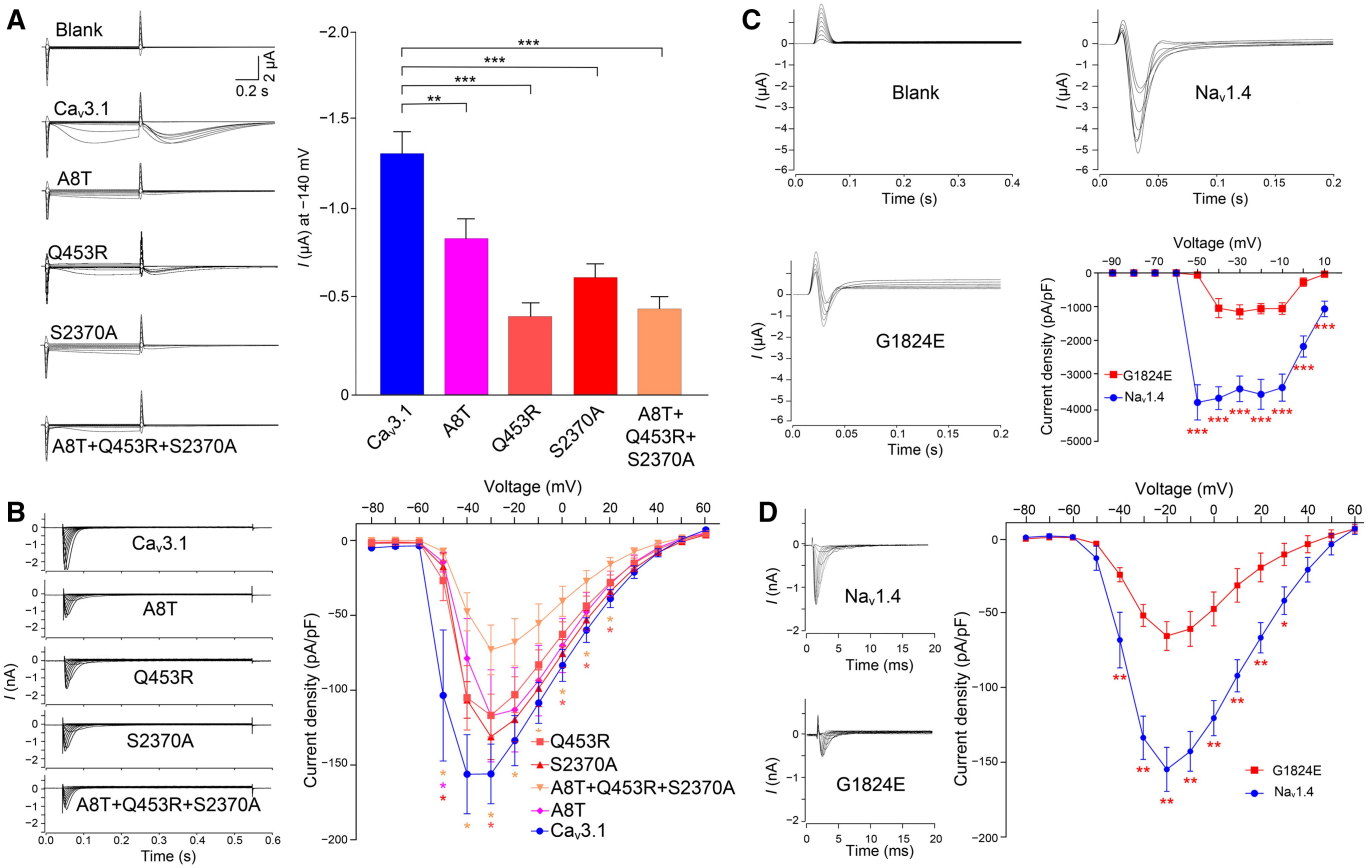
Physiological changes in ion channels with limestone langur-specific AA substitutions **A**. Current traces from *Xenopus laevis* oocytes expressing wild-type Ca_v_3.1 as well as Ca_v_3.1-A8T, Ca_v_3.1-Q453R, Ca_v_3.1-S2370A, Ca_v_3.1-A8T+Q453R+S2370A variants, with the statistics of the current diversity at −140 mV (*n* = 10 biological replicates; Wilcoxon two-sided test; **, *P* < 0.01; ***, *P* < 0.001). **B**. Current traces from HEK-293T cells expressing wild-type Ca_v_3.1 and variants, with the current density–voltage curves (*n* = 6; Wilcoxon two-sided test; *, *P* < 0.05). **C**. Current traces from *Xenopus laevis* oocytes expressing wild-type Na_v_1.4 and Na_v_1.4-G1824E variant, with the statistics of the current amplitudes from multiple recordings (*n* ≥ 6; Wilcoxon two-sided test; ***, *P* < 0.001). **D**. Current traces from HEK-293T cells expressing wild-type Na_v_1.4 and Na_v_1.4-G1824E variant, with the current density–voltage curves (*n* = 6; Wilcoxon two-sided test; *, *P* < 0.05; **, *P* < 0.01).

Among the 10 voltage-gated calcium channels, Ca_v_3.1 (encoded by *CACNA1G*) exhibited the highest number of limestone langur-specific AA substitutions and was identified as a REG-encoded protein. Thus, Ca_v_3.1 was selected to assess potential physiological changes caused by these AA substitutions. Variants in Ca_v_3.1 are known to be associated with various channelopathies in humans, such as autosomal-dominant cerebellar ataxia (R1715H), cerebellar atrophy, and brainstem defects (M1574L) [28,29]. In limestone langurs, three AA substitutions (A8T, Q453R, and S2370A) were consistently present. To examine their biophysical properties, we performed *in vitro* transcription of the wild-type and mutant *CACNA1G* genes and injected the capped RNAs (cRNAs) into *Xenopus laevis* oocytes. The inward currents produced by the mutated channels (Ca_v_3.1-A8T, Ca_v_3.1-Q453R, Ca_v_3.1-S2370A, and Ca_v_3.1-A8T+Q453R+S2370A) were significantly lower than that generated by the wild-type Ca_v_3.1, as measured by two-electrode voltage-clamp analysis (**Figure 6**A). We further validated these results by transiently expressing the wild-type and mutant channels in HEK-293T cells and performing whole-cell voltage-clamp analysis. Current amplitudes were significantly reduced in HEK-293T cells expressing the mutated channels (Wilcoxon two-sided test, *P* < 0.05) (Figure 6B), indicating that Ca_v_3.1-A8T, Ca_v_3.1-Q453R, Ca_v_3.1-S2370A, and Ca_v_3.1-A8T+Q453R+S2370A result in decreased Ca^2+^ currents (Figure 6A and B).

Beyond PSG- and REG-encoded proteins, lineage-specific AA mutations in proteins involved in adaptively evolved pathways may also have functional consequences [30]. We selected Na_v_1.4 (encoded by *SCN4A*) to test the effect of the G1824E substitution on the channel activity (*I*_Na_), as all limestone langurs possess this specific AA substitution. Na_v_1.4 plays a critical role in muscle membrane excitability and contraction, and it is the predominant sodium channel in adult skeletal muscle, which accounts for a major component of the total Na^+^ [31]. Mutations in the *SCN4A* have been linked to various neuromuscular disorders in humans, including myotonia (G1306E), hyperkalemic periodic paralysis (T704M), and myasthenic myopathy (R1454W) [32–34]. It is reasonable to hypothesize that G1824E might play a vital role in skeletal muscle performance, aiding limestone langurs in climbing, and adapting to harsh environments. We examined the biophysical properties of Na_v_1.4 with and without the substitution G1824E in *Xenopus laevis* oocytes and HEK-293T cells, and measured *I*_Na_ using voltage-clamp technologies. The inward current produced by Na_v_1.4-G1824E was significantly lower than that generated by the wild-type Na_v_1.4 (Wilcoxon two-sided test, *P* < 0.05) (Figure 6C and D).

### The E94D substitution in melanocortin 1 receptor contributes to the darker fur color of limestone langurs

**Figure 7:**
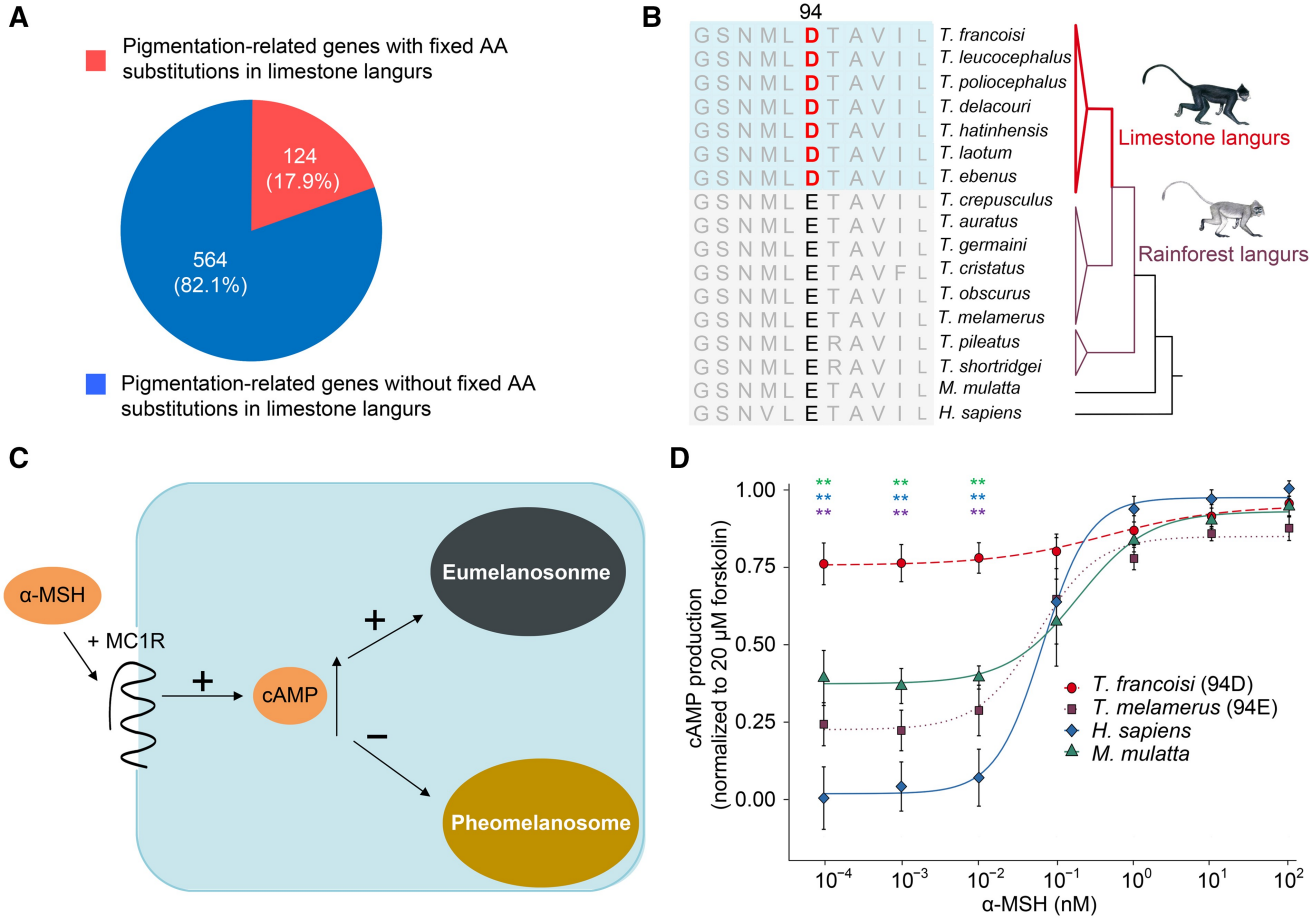
Functional experiments of MC1R **A**. Of the 688 pigmentation-related genes, 124 (17.9%) contain AA substitutions fixed across all limestone langurs. **B**. Partial sequence alignment of MC1Rs of langurs and other primates. **C**. The role of MC1R in regulating melanin synthesis during pigmentation. High basal MC1R signaling increases cAMP production, leading to higher eumelanin synthesis. Lower cAMP levels lead to increased pheomelanin synthesis. **D**. Generation of cAMP responding to α-MSH treatment in HEK-293T cells expressing MC1Rs from *T. francoisi*, *T. melamerus*, *H. sapiens*, and *M. mulatta* (*n* = 3; Wilcoxon two-sided test; **, *P* < 0.01). MC1R, melanocortin 1 receptor; cAMP, cyclic adenosine monophosphate; α-MSH, α-melanocyte-stimulating hormone.

Phenotypically, limestone langurs typically exhibit black fur, whereas rainforest langurs have predominantly gray or brownish coats (Figure 2A). To elucidate the genetic mechanism underlying these coat color differences, we investigated protein-coding sequence variants in 688 pigmentation-related genes [35]. Among these, 124 genes (17.9%) had AA substitutions fixed across all limestone langurs (**Figure 7**A; Table S8). For example, an AA substitution (E94D) in the melanocortin 1 receptor (*MC1R*) gene was present in all investigated limestone langurs but absent in rainforest langurs (Figure 7B).

MC1R plays a key role in pigmentation by regulating melanin production and distribution in animal skin and hair. Along with its second messenger cyclic adenosine monophosphate (cAMP), MC1R controls the switch between the synthesis of eumelanin and pheomelanin, which influences hair, skin, and eye color. High levels of cAMP signaling cause increased eumelanin synthesis and make the hair darker (Figure 7C) [36,37]. To further assess the impact of the E94D substitution on melanin synthesis, we conducted *in vitro* cAMP assays using MC1R-94E and MC1R-94D variants. Intracellular cAMP production was measured in response to varying concentrations of α-melanocyte-stimulating hormone (α-MSH) in MC1Rs from four primate species (*H. sapiens*, *M. mulatta*, *T. francoisi*, and *T. melamerus*). The E94D substitution in limestone langurs (*T. francoisi*) resulted in significantly higher basal cAMP production compared to the rainforest langur (*T. melamerus*) and other primates (*H. sapiens* and *M. mulatta*) (Wilcoxon two-sided test, *P* < 0.01) (Figure 7D). This suggests that MC1R E94D substitution contributes to the darker fur color observed in limestone langurs.

## Discussion

Advances in DNA sequencing and genome assembly have enabled the generation of significantly improved reference genomes, particularly in terms of contiguity and completeness, allowing for better annotations and more accurate genetic and evolutionary interpretations [14]. The high-quality genome of *T. francoisi*, combined with broad sampling across *Trachypithecus* spp., has enabled us to reconstruct the phylogenetic history of these langurs and revealed several adaptive mechanisms, particularly in limestone langurs, which inhabit a challenging environment.

Our divergence time estimates suggest that limestone langurs diverged from other *Trachypithecus* spp. 2.44–3.04 MYA, coinciding with the rapid development of local karst formations in the early Quaternary (∼ 2.50 MYA) [38]. Similar to other organisms living in karst environments, but in contrast to rainforest langurs, limestone langurs have evolved to adapt to an inherently high intake of minerals and metal ions [5,6]. Limestone langurs have been observed drinking from karst water sources, where the concentrations of Na^+^, K^+^, Ca^2+^, and Mg^2+^ and pH values are significantly higher than those of nearby rivers (Figure S9A) [39,40]. Data from breeding centers and zoos further show that blood concentrations of Na^+^, K^+^, Ca^2+^, Mg^2+^, and P (phosphorus) are markedly higher in *T. francoisi* compared to *H*. *sapiens*, *M*. *mulatta*, and *M*. *fascicularis* (Figure S9B). Therefore, the high blood ion concentration is one of the most remarkable biological characteristics of limestone langurs, which tends to increase extracellular ion concentrations in various tissues. Consistent with these observations, the significantly elevated d*N*/d*S* ratios of genes encoding Na^+^, K^+^, and Ca^2+^ channels, along with other ion channel-related genes, suggest that these genes evolved in response to environmental pressures in limestone langurs.

This study, along with previous work, has confirmed that specific AA mutations in ion channels (Na_v_1.4-G1824E, Ca_v_1.2-K1905R [6], Ca_v_3.1-A8T, Ca_v_3.1-Q453R, and Ca_v_3.1-S2370A) reduce channel activities *in vitro*. Na_v_1.4, highly expressed in adult skeletal muscle, is responsible for the bulk of the inward sodium current that generates the muscle action potential, crucial for muscle relaxation and contraction [34]. Normally, excessive sodium currents delay muscle relaxation [41], which can impair movement. Given the significantly higher blood sodium concentration in limestone langurs compared to other primates (Figure S9B), we hypothesize that limestone langurs have evolved adaptive mechanisms to cope with the karst environment. The fixed Na_v_1.4-G1824E mutation attenuates inward sodium currents, reducing current density and potentially enhancing skeletal muscle performance, which may assist in climbing the rocky terrain of karst habitats. Ca_v_3.1 is predominantly expressed in the central nervous system, where Ca^2+^ influx through this channel facilitates axonal and dendritic outgrowth and regulates cell death [42]. Ca_v_3.1 also modulates membrane potential and calcium entry into neurons [43]. Normally, mutations in the Ca_v_3.1 cause damage to the central nervous system, impairing limb coordination. Moreover, the G782S mutation in Ca_v_3.2, found in all limestone langurs, is a pathogenic mutation (G784S) in humans, causing childhood absence epilepsy [44]. This mutation results in slower activation, faster inactivation of Ca_v_3.2, and decreased Ca^2+^ current [44]. In general, AA mutations in ion channels in limestone langurs appear to alter ion currents, potentially increasing their tolerance to high ion concentrations.

The functional consequences of ion channel mutations in limestone langurs provide insights into human channelopathies. Channelopathies are disorders caused by dysfunctions of ion channels, often due to inherited mutations or abnormal gene expression. Mutations in ion signaling pathways are frequently associated with channelopathies and other human diseases. Most limestone langur-specific AA mutations occur in the intracellular loops between transmembrane domains and termini (Figure 4A–C), regions known to interact with regulatory proteins [45–47]. Human channelopathies often involve mutations in the I–II and II–III intracellular loops of sodium and calcium channels, underscoring the functional importance of these regions [45,47]. Among the 58 AA mutations in the Na^+^, K^+^, and Ca^2+^ channels found in limestone langurs but not in rainforest langurs, 53 are present in human populations based on the databases of 1000 Genomes Project (1KGP), Genome Aggregation Database (gnomAD), and Trans-Omics for Precision Medicine (TOPMed). These mutations are at extremely low frequencies (< 5 × 10^−4^) in human populations, indicating that these sites are highly conserved and under negative selection in humans. Moreover, 10 of these substitutions which are listed in the human ClinVar database are associated with conditions such as epilepsy, hemiplegic migraine, early infantile epileptic encephalopathy, Brugada syndrome, and hereditary sensory and autonomic neuropathy. The biophysical changes caused by these mutations in ion channels should be further investigated in human populations to better understand their roles in channelopathies.

Intracellular signaling mediated by Na^+^, K^+^, Ca^2+^, and other ion channels is interconnected through several molecular pathways [42]. For instance, vertebrate cardiac muscle fibers exhibit action potential lasting 100–500 ms, essential for myocardial contraction. The duration is sustained by a balance between an initial inward Na^+^ current, a subsequent slow Ca^2+^ current, and a diminishing outward K^+^ current (Figure 5G) [45]. The ionic basis of cell action potential is complex and varies among cell types, but it universally involves integrated dynamics of Na^+^, K^+^, and Ca^2+^ currents. Our coevolution analysis supports this integrated framework, revealing evolutionary integrity among ion channels (Figure 5E–G). Parallel sequencing of ion channel genes in humans with and without idiopathic epilepsy has demonstrated that the deleterious effects of ion channel mutations often depend on variants in other associated channels [13]. Our findings in limestone langurs suggest an oligogenic/polygenic mechanism underlying ion channel evolution, rather than a monogenic pattern. However, studies exploring network models that integrate ion channel genotypes and phenotypes remain limited and that integrate feedback loops and regulatory networks are more interconnected than previously recognized, potentially reflecting coevolutionary processes.

The dynamic equilibrium of metal ions (Na^+^, K^+^, Ca^2+^, *etc.*) in organisms is essential for normal physiological processes, but these ions become toxic when their concentrations exceed a certain threshold [48]. Excessive metal ions can impair DNA synthesis and replication, ultimately leading to cancer and other diseases [49]. Consistent with this, we detected adaptive evolutionary signals in genes related to DNA damage response/repair in limestone langurs, such as *RNF168*, *CYB5R4*, and *NUB1* (Figure 3A, C, and D). RNF168 interacts with ubiquitinated linker histones at DNA damage sites, facilitating accurate DNA repair [50,51]. CYB5R4 may participate in the DNA damage response by modulating PP2A family phosphatases [52], and NUB1 supports DNA repair by promoting the degradation of FAT10, a protein linked to chromosomal instability [53]. These findings suggest that limestone langurs, like other organisms exposed to high levels of environmental metal ions, have evolved not only ion channel adaptations but also mechanisms to mitigate DNA damage, reflecting the environmental pressures of karst habitats. It is reasonable to infer that the environmental stress of karst habitats has influenced the DNA stability of limestone langurs and that the detected REGs and PSGs related to DNA damage response/repair have regulated the fine-tuned adaptive balance.

Coat color is one of the most visible traits in animals and has been extensively studied in the context of environmental adaptation and evolutionary change [54]. Limestone langurs exhibit primarily black coats with occasional white/yellow patches on the head, rump, or tail. The black coloration of limestone langurs was speculated to be an adaptive and protective coloration in karst habitats [10,55]. MC1R is a critical regulator of pigmentation in vertebrates, and MC1R variants are often associated with body color polymorphism [56]. We identified the E94D substitution in MC1R in all limestone langurs. A similar substitution, E94K, is responsible for dark plumage in species such as bananaquit, quail, and chickens [57,58]. Our functional experiments showed that the MC1R-E94D leads to higher constitutive activation in limestone langurs compared to rainforest langurs. It is tempting to speculate that this substitution has contributed to the dark fur color in limestone langurs and that the 94th AA is key to MC1R function in vertebrates. Additional fixed mutations in pigmentation-related proteins (*e.g.*, EDN3, EDNRB, KIT, and PMEL) may elevate endothelin signaling, leading to increased eumelanin production. These findings offer a potential explanation for the morphological adaptations of limestone langurs and provide new insights into body color variation in other vertebrates.

## Conclusion

This study illustrates how improving a genome assembly enhances the interpretation of evolutionary processes. The high-quality genome assembly and extended sampling of *Trachypithecus* species have provided the necessary resources to uncover and validate the adaptations of limestone langurs. Our study revealed significant adaptive evolution in genes related to ion channels and selection signals in DNA damage response/repair pathways, which likely enable limestone langurs to survive in karst environments characterized by metal toxicity. In addition to physiological adaptations, we also identified genetic underpinnings of phenotypic adaptations, such as darker coat color in limestone langurs. Collectively, these findings underscore the complexity of the interactions between organisms and their environments.

The evolutionary genomics of limestone langurs provides essential insights for developing scientifically informed conservation strategies. *In situ* and *ex situ* conservation are the two primary approaches to preserving wildlife and maintaining genetic diversity. Our findings emphasize the importance of *in situ* conservation for species in specialized habitats, such as limestone langurs in karst environments. Moreover, *ex situ* conservation efforts must consider the genomic background of species like limestone langurs. Adaptive substitutions that enhance survival in metal-rich environments may become deleterious when animals are relocated to environments with lower metal ion concentrations. These substitutions affect the functions of proteins related to ion channels, DNA damage response, and other pathways, and may not be beneficial in captive populations with lower metal ion intake. In this perspective, the genotype–environment interactions are of major significance to the genetic management of endangered species, because the fitness of translocated individuals cannot be predicted if there are significant, but poorly understood, genotype–environment interactions.

Soil salinization, which increases ion concentrations in soil and leads to metal toxicity, is a major global environmental challenge that will likely worsen due to climate change, further threatening biodiversity [59]. Our findings in limestone langurs might provide insights into the adaptive potential of other organisms currently challenged by soil salinization and the persistence of the biodiversity affected by global changes. The adaptation of limestone langurs to karst habitats occurred over at least one million years and involved hundreds of genes. This suggests potential target genes and pathways that could be affected by metal toxicity in other species facing similar environmental challenges. Studies of adaptation in natural populations allow researchers to uncover how organisms adapt genomically, phenotypically, and physiologically to their environments, providing practical implications for conservation.

## Materials and methods

### Sample and data collection

Samples were collected from 48 individuals representing 7 species of limestone langurs and 8 species of rainforest langurs (Table S3). Of these, 25 genomes were newly sequenced, and the remaining 23 genomes were retrieved from the National Center for Biotechnology Information (BioProject: PRJNA488530) generated in a previous study [6] (Table S3). All sample collections complied with institutional and legal regulations. Tissue and skin samples were obtained from remains provided by local museums and nature reserves. Tissue samples were from naturally deceased individuals in nature reserves and zoos and were preserved at −20°C or −80°C. Skin samples were stored dry. To prevent contamination, DNA extraction was performed in a clean beach with positive air pressure, and staff used full-body protection, including masks, gloves, protective clothing, and caps. All work surfaces and equipment were cleaned with 10% bleach and sterile water, followed by exposure to ultraviolet (UV) light for 30 min before handling. UV irradiation was also applied for 30 min between uses on all working areas, tools, and consumables. Genomic DNA was extracted using a QIAamp DNA Mini Kit (Catalog No. 51306, QIAGEN, Shanghai, China) following the manufacturer’s protocol. DNA quality and degradation were assessed using an Agilent 2100 Bioanalyzer (Agilent Technologies, Santa Clara, CA). The *T. francoisi* genome was sequenced using single-molecule real-time (SMRT) HiFi technology (Pacific BioSciences, Menlo Park, CA). Genomic DNA (8 μg) was sheared using g-TUBE (Catalog No. 520079, Covaris, Woburn, MA) and concentrated with AMPure PB magnetic beads (Catalog No. 100-265-900, Pacific BioSciences). SMRTbell libraries were constructed using the PacBio HiFi SMRTbell prep kit 3.0 (Catalog No. 102-182-700, Pacific BioSciences), following the manufacturer’s instructions. Libraries were size-selected for molecules of ∼ 20 kb using a BluePippin system (Sage Science, Beverly, MA). Primers were annealed, and SMRTbell templates were bound to polymerases using a DNA/Polymerase Binding Kit (Catalog No. 100-372-700, Pacific BioSciences). Sequencing was performed on a PacBio Sequel IIe for 10 h. Whole-genome resequencing of other langur individuals was performed on the Illumina HiSeq 2500 platform (Illumina, San Diego, CA).

### Genome assembly, annotation, resequencing, and variant calling

Genomic DNA was extracted from tissue samples through the QIAGEN DNeasy Blood & Tissue Kit (Catalog No. 69504, QIAGEN). Five libraries were constructed to obtain long PacBio HiFi reads. HiFi sequences were assembled using hifiasm (v0.61.1-r375) with default parameters [60]. Chromosome-scale scaffolding was performed using 3D-DNA (v201008, https://github.com/aidenlab/3d-dna) [61], based on chromatin interactions obtained through *in situ* Hi-C data. Genome synteny between langur, macaque (Mmul_10), and human (GRCh38.p13) was analyzed using LASTZ [62] with the parameters: M = 254, K = 4500, L = 3000, Y = 15,000, and T = 2, filtering alignments less than 1 kb. Further synteny analyses were refined using MUMmer (v4.0.0rc1) [63] and visualized using Circos (v0.69-8) [64] with default parameters (Figure S2). Scaffold and contig N50 values were calculated using QUAST (v5.2.0) [65]. Assembly completeness was assessed using the mammalia odb9 database in BUSCO (v5.4.2) [15] with default parameters. Protein-coding genes were predicted using MAKER (v3.01.04) [66] based on transcriptomic data, homologous genes, and *de novo* predictions. Functional annotation was performed using eggNOG-mapper online [67]. Mt-genome assembly and annotation were conducted using MitoZ (v3.6) with the “all” module [68]. The process of genomic DNA extraction, genome resequencing, and SNP calling for langur individuals followed the procedures described by Liu and his colleagues [6]. To assess DNA damage levels, final binary alignment map (BAM) files for each sample were analyzed using mapDamage2.0 [16].

### Orthologous gene family clustering and phylogenetic tree construction

Protein-coding gene sequences from *T. francoisi* and nine primates (*T. melamerus*, *R. roxellana*, *R. bieti*, *M. fascicularis*, *M. mulatta*, *P. anubis*, *C. atys*, *P. troglodytes*, and *H. sapiens*) were used for orthologous gene family clustering. Expansion and contraction of orthologous gene families were determined by comparing cluster size differences between the ancestral *T. francoisi* and each of aforementioned nine primates. The longest transcript was selected to represent each gene. First, the Basic Local Alignment Search Tool for Proteins (BLASTP; v2.11.0+) [69] was used to perform all-against-all comparison of proteins with a cutoff E-value < 1E−5. Genes were then clustered into gene families using the updated OrthoMCL pipeline [18]. From these, the protein sequences of 11,529 one-to-one orthologs (Figure S4) were extracted and aligned using MAFFT (v7.520) and PRANK [70,71]. Gene family expansion/contraction analysis was conducted by comparing the gene family sizes between *T. francoisi* and other aforementioned nine primates using CAFÉ (v4.2.1) [72].

To obtain the protein-coding sequences of *Trachypithecus* spp., individual genomes were generated based on fixed SNP data using bcftools consensus option (bcftools consensus personalized.fixed.snp.vcf.gz -o personalized.genome.fa) [73]. Coding sequences of the 11,529 one-to-one orthologs were extracted for each langur individual and were integrated with datasets from the ten primate species. Phylogenetic analyses were conducted using the ML algorithm in RAxML (v8) with the GTRGAMMA substitution model and 1000 nonparametric bootstrap replicates based on fourfold degenerate sites of the protein-coding sequences [74]. Divergence time was estimated based on the Markov chain Monte Carlo (MCMC) algorithm using MCMCTree in PAML (v4) and IQ-TREE2 [19,75]. Four fossil-calibrated nodes were used for divergence estimation [76]: (1) most recent common ancestor (MRCA) of *Homo* and *Pan*: 4.63–15.00 MYA; (2) MRCA of *Papio* and *Macaca*: 5.33–12.51 MYA; (3) MRCA of Cercopithecidae: 12.47–25.24 MYA; and (4) MRCA of Catarrhini: 25.19–35.10 MYA (red dots in Figure S6A).

For mt-genome analysis, an ML tree was constructed using RAxML (v8) [74] with the GTRGAMMA model, chosen through rapid bootstrapping and 1000 bootstrap replicates. Divergence time was estimated using BEAST (v2) [77] with two independent runs of 5,000,000 generations and a burn-in of 1,000,000 generations. Seven calibration nodes from published fossil records of platyrrhine primates [76] were applied: (1) MRCA of *Homo* and *Pan*: 4.63–15.00 MYA; (2) MRCA of Hominidae: 12.30–25.24 MYA; (3) MRCA of *Papio* and *Macaca*: 5.33–12.51 MYA; (4) MRCA of Cercopithecinae: 6.50–15.00 MYA; (5) MRCA of Colobinae: 8.13–15.00 MYA; (6) MRCA of Cercopithecidae: 12.47–25.24 MYA; and (7) MRCA of Catarrhini: 25.19–35.10 MYA (red dots in Figure S6B). Convergence of the runs was assessed using Tracer (v1.7) [78].

### Identification of REGs and PSGs in *T. francoisi*

The 11,529 one-to-one orthologous protein-coding gene sequences of *T. francoisi* and nine primate species were corrected using the Sliding Window Alignment Masker for PAML (SWAMP; v1.0) [79] to filter out abnormally high nonsynonymous substitutions with default thresholds. Rapid evolution and positive selection signals for genes in the *T. francoisi* branch were detected using branch and branch-site models using codeML in PAML (v4) software [19] by estimating the d*N*/d*S* ratios, setting *T. francoisi* as foreground branch and other nine primates as background branches. Gene set enrichment analysis (GSEA) for REGs and PSGs was performed using Metascape online [80] with default settings.

### Estimation of evolutionary rates of ion channel genes in *Trachypithecus* spp.

To further investigate the molecular evolution of ion channel genes within the genus *Trachypithecus*, protein-coding sequences of ion channel genes were extracted from 15 *Trachypithecus* species (one individual randomly selected per species). Changes in evolutionary ratios (d*N*/d*S*) between limestone (foreground branch) and rainforest (background branch) langurs were calculated with codeML in PAML (v4) [19] using the branch model.

### Coevolution analyses

Coevolution analyses between ion channel pairs were performed using two approaches: phylogenetic profiling and phylogenetic tree similarity [81]. First, coevolving lineages were predicted using PrePhyloPro online [25], based on phylogenetic profiling. The datasets comprised 57,114 positive and 65,520 negative linkages provided by PrePhyloPro, which were used as controls. Then, MirrorTree (v1) [24] was used to calculate the similarity of phylogenetic trees among proteins based on protein sequences. Positive and negative control datasets from PrePhyloPro [25] and the human protein binary interaction interactome [27] were used for the MirrorTree [24] analysis.

### Comparative analysis of pigmentation-related genes

Protein-coding sequences of 688 pigmentation-related genes were extracted from limestone and rainforest langur species based on SNP information. Genes with limestone langur-specific AA mutations (fixed in all limestone langur individuals) were manually validated.

### Laboratory methods

#### Two-electrode voltage-clamp analysis

The complementary DNAs (cDNAs) of human *CACNA1G* (encoding Ca_v_3.1) and *SCN4A* (encoding Na_v_1.4) were synthesized by Sangon Biotech (Shanghai, China), using the pGEMHE vector as the plasmid backbone. Mutations were introduced using a Site-Directed Mutagenesis Kit (Catalog No. KM101, TIANGEN, Beijing, China), and all constructs were verified by sequencing. Oocytes were injected with 32 nl of cRNAs at a concentration of 500 ng/µl or with 32 nl of RNase-free water (control oocytes), followed by incubation at 16°C for 3 days before electrophysiological recordings. Electrophysiological recordings were performed according to Pan et al. [82], with at least 10 oocytes tested for each channel (with or without mutations). All data are presented as mean ± standard deviation (SD), with statistical significance set at *P* < 0.05.

#### Whole-cell voltage-clamp recordings

The cDNAs of wild-type *CACNA1G* (encoding Ca_v_3.1) and *SCN4A* (encoding Na_v_1.4) as well as their mutants were cloned into pCDH-CMV-MCS-copGFP vector (Catalog No. CD523A-1, System Biosciences, Palo Alto, CA). HEK-293T cells were then transiently transfected with 5 µg of vectors carrying wild-type or mutant genes using Lipofectamine 2000 (Catalog No. 11668-019, Thermo Fisher Scientific, Waltham, MA). Each transfection was performed in triplicate. Western blot analyses were conducted in parallel for wild-type and mutant channels (Figure S10). Whole-cell voltage-clamp recordings were performed for wild-type and mutant channels (*n* = 6 for each vector), following the procedure described by Trachsel and his colleagues [83].

#### Functional analysis of MC1Rs of limestone and rainforest langurs

The constitutive activity and α-MSH agonist-induced response of the MC1R variants were evaluated *in vitro* as described by Yan and his colleagues [84]. Briefly, 1 ng of pcDNA3.3-Flag-MC1R vector was transiently transfected into HEK-293T cells. After 24 h of incubation at 37°C, a cAMP-Gs Dynamic assay was performed. Cells were stimulated with varying concentrations of α-MSH (from 0.0001 to 100 nM), and fluorescence signals were detected using the FlexStation 3 Microplate Reader (Molecular Devices Corporation, Sunnyvale, CA). Transfection efficiency was normalized by treating cells with 20 µM forskolin, which was subsequently adjusted to a final concentration of 10 µM. MC1R expression vectors from rhesus macaque (*M. mulatta*) and human (*H. sapiens*) served as positive controls, and the empty pcDNA3.3 vector was used as a mock control. The cAMP assay was conducted in triplicate for each MC1R expression vector.

## Ethical statement

Tissue samples were obtained from museum specimens. No experiment was conducted on living animals. The Ethics Committee of College of Life Science, Capital Normal University, China approved this study (Approval No. IACUC NO.2021032).

## Supplementary Material

qzaf007_Supplementary_Data

## Data Availability

The HiFi sequencing data and the raw resequencing data for the newly generated langur genomes have been submitted to the Genome Sequence Archive [85] at the National Genomics Data Center (NGDC), China National Center for Bioinformation (CNCB) (GSA: CRA007753; BioProject: PRJCA010872), and are publicly accessible at https://ngdc.cncb.ac.cn/gsa. The genome assemblies have been submitted to the Genome Warehouse [86] at the NGDC, CNCB (GWH: GWHBOZZ00000000), and are publicly accessible at https://ngdc.cncb.ac.cn/gwh.

## CRediT author statement

**Zhijin Liu:** Conceptualization, Methodology, Software, Supervision, Project administration, Funding acquisition, Writing – original draft, Writing – review & editing. **Xiongfei Zhang:** Data curation, Investigation, Resources, Validation, Formal analysis. **Peipei Wang:** Investigation, Resources, Validation, Writing – original draft, Writing – review & editing. **Minheng Hong:** Investigation, Resources, Validation. **Xiaochan Yan:** Investigation, Resources, Validation. **Xiaoqiu Qi:** Methodology, Software, Writing – original draft, Writing – review & editing. **Qian Zhao:** Formal analysis. **Zhenghao Chen:** Formal analysis. **Huajian Nie:** Formal analysis. **Hui Li:** Formal analysis. **Ziwen Li:** Formal analysis. **Liye Zhang:** Formal analysis. **Jiwei Qi:** Formal analysis. **Chaolei He:** Formal analysis. **Nguyen Van Truong:** Resources. **Minh D. Le:** Resources. **Tilo Nadler:** Resources. **Hiroo Imai:** Resources, Data Curation, Validation. **Christian Roos:** Writing – original draft, Supervision, Project administration, Funding acquisition. **Ming Li:** Writing – original draft, Supervision, Project administration, Funding acquisition. All authors have read and approved the final manuscript.

## ORCID

0009-0006-9550-5803 (Zhijin Liu)

0009-0000-0253-3976 (Xiongfei Zhang)

0009-0004-2474-4327 (Peipei Wang)

0009-0003-5843-9323 (Minheng Hong)

0009-0004-1041-9718 (Xiaochan Yan)

0009-0009-2130-9844 (Xiaoqiu Qi)

0009-0001-8157-8300 (Qian Zhao)

0009-0004-0846-4683 (Zhenghao Chen)

0009-0008-1935-9685 (Huajian Nie)

0009-0009-5774-5410 (Hui Li)

0009-0002-8556-0949 (Ziwen Li)

0000-0002-8829-0588 (Liye Zhang)

0009-0000-2023-091X (Jiwei Qi)

0009-0007-1250-9428 (Chaolei He)

0000-0003-2004-6658 (Nguyen Van Truong)

0000-0002-2953-2815 (Minh D. Le)

0009-0002-2421-572X (Tilo Nadler)

0000-0003-0729-0322 (Hiroo Imai)

0000-0003-0190-4266 (Christian Roos)

0000-0001-5689-6270 (Ming Li)

## References

[1] Kuderna LFK , Gao H, Janiak MC, Kuhlwilm M, Orkin JD, Bataillon T, et al A global catalog of whole-genome diversity from 233 primate species. Science 2023;380:906–13. 37262161 10.1126/science.abn7829 PMC12120848

[2] Shao Y , Zhou L, Li F, Zhao L, Zhang BL, Shao F, et al Phylogenomic analyses provide insights into primate evolution. Science 2023;380:913–24. 37262173 10.1126/science.abn6919

[3] Goldscheider N , Chen Z, Auler AS, Bakalowicz M, Broda S, Drew D, et al Global distribution of carbonate rocks and karst water resources. Hydrogeol J 2020;28:1661–77.

[4] Shen Y , Yu Y, Lucas-Borja ME, Chen F, Chen Q, Tang Y. Change of soil K, N and P following forest restoration in rock outcrop rich karst area. Catena 2020;186:104395.

[5] Cao Y , Almeida-Silva F, Zhang WP, Ding YM, Bai D, Bai WN, et al Genomic insights into adaptation to karst limestone and incipient speciation in East Asian *Platycarya* spp. (Juglandaceae). Mol Biol Evol 2023;40:msad121. 37216901 10.1093/molbev/msad121 PMC10257982

[6] Liu Z , Zhang L, Yan Z, Ren Z, Han F, Tan X, et al Genomic mechanisms of physiological and morphological adaptations of limestone langurs to karst habitats. Mol Biol Evol 2020;37:952–68. 31846031 10.1093/molbev/msz301

[7] Zhou Y , Fan W, Zhang H, Zhang J, Zhang G, Wang D, et al *Marsdenia tenacissima* genome reveals calcium adaptation and tenacissoside biosynthesis. Plant J 2023;113:1146–59. 36575579 10.1111/tpj.16081

[8] Tao J , Feng C, Ai B, Kang M. Adaptive molecular evolution of the two-pore channel 1 gene TPC1 in the karst-adapted genus *Primulina* (Gesneriaceae). Ann Bot 2016;118:1257–68. 27582362 10.1093/aob/mcw168 PMC5155596

[9] Russell AM , Anthony BR, Don EW. Handbook of the mammals of the world: 3. primates. Barcelona: Lynx Ediciones; 2013.

[10] Huang C , Wu H, Zhou Q, Li Y, Cai X. Feeding strategy of François’ langur and white-headed langur at Fusui, China. Am J Primatol 2008;70:320–6. 17924424 10.1002/ajp.20490

[11] Galle OK. Chemical analysis of some standard carbonate rocks. Chem Geol 1969;5:143–6.

[12] Kamilar JM. Interspecific variation in primate countershading: effects of activity pattern, body mass, and phylogeny. Int J Primatol 2009;30:877–91.

[13] Klassen T , Davis C, Goldman A, Burgess D, Chen T, Wheeler D, et al Exome sequencing of ion channel genes reveals complex profiles confounding personal risk assessment in epilepsy. Cell 2011;145:1036–48. 21703448 10.1016/j.cell.2011.05.025 PMC3131217

[14] Jung H , Ventura T, Chung JS, Kim WJ, Nam BH, Kong HJ, et al Twelve quick steps for genome assembly and annotation in the classroom. PLoS Comput Biol 2020;16:e1008325. 33180771 10.1371/journal.pcbi.1008325 PMC7660529

[15] Simão FA , Waterhouse RM, Ioannidis P, Kriventseva EV, Zdobnov EM. BUSCO: assessing genome assembly and annotation completeness with single-copy orthologs. Bioinformatics 2015;31:3210–2. 26059717 10.1093/bioinformatics/btv351

[16] Jónsson H , Ginolhac A, Schubert M, Johnson PL, Orlando L. mapDamage2.0: fast approximate Bayesian estimates of ancient DNA damage parameters. Bioinformatics 2013;29:1682–4. 23613487 10.1093/bioinformatics/btt193 PMC3694634

[17] Sawyer S , Krause J, Guschanski K, Savolainen V, Pääbo S. Temporal patterns of nucleotide misincorporations and DNA fragmentation in ancient DNA. PLoS One 2012;7:e34131. 22479540 10.1371/journal.pone.0034131 PMC3316601

[18] Glover N , Dessimoz C, Ebersberger I, Forslund SK, Gabaldón T, Huerta-Cepas J, et al Advances and applications in the Quest for Orthologs. Mol Biol Evol 2019;36:2157–64. 31241141 10.1093/molbev/msz150 PMC6759064

[19] Yang Z. PAML 4: phylogenetic analysis by maximum likelihood. Mol Biol Evol 2007;24:1586–91. 17483113 10.1093/molbev/msm088

[20] Cornelis MC , Fornage M, Foy M, Xun P, Gladyshev VN, Morris S, et al Genome-wide association study of selenium concentrations. Hum Mol Genet 2015;24:1469–77. 25343990 10.1093/hmg/ddu546 PMC4321444

[21] Bassil J , Naveau A, Bueno M, Razack M, Kazpard V. Leaching behavior of selenium from the karst infillings of the Hydrogeological Experimental Site of Poitiers. Chem Geol 2018;483:141–50.

[22] Xiao K , Lu L, Tang J, Chen H, Li D, Liu Y. Parent material modulates land use effects on soil selenium bioavailability in a selenium-enriched region of southwest China. Geoderma 2020;376:114554.

[23] Olivieri M , Cho T, Álvarez-Quilón A, Li K, Schellenberg MJ, Zimmermann M, et al A genetic map of the response to DNA damage in human cells. Cell 2020;182:481–96.e21. 32649862 10.1016/j.cell.2020.05.040 PMC7384976

[24] Ochoa D , Pazos F. Studying the co-evolution of protein families with the MirrorTree web server. Bioinformatics 2010;26:1370–1. 20363731 10.1093/bioinformatics/btq137

[25] Niu Y , Liu C, Moghimyfiroozabad S, Yang Y, Alavian KN. PrePhyloPro: phylogenetic profile-based prediction of whole proteome linkages. PeerJ 2017;5:e3712. 28875072 10.7717/peerj.3712 PMC5578374

[26] Ranwez V , Delsuc F, Ranwez S, Belkhir K, Tilak MK, Douzery EJ. OrthoMaM: a database of orthologous genomic markers for placental mammal phylogenetics. BMC Evol Biol 2007;7:241. 18053139 10.1186/1471-2148-7-241 PMC2249597

[27] Luck K , Kim DK, Lambourne L, Spirohn K, Begg BE, Bian W, et al A reference map of the human binary protein interactome. Nature 2020;580:402–8. 32296183 10.1038/s41586-020-2188-x PMC7169983

[28] Coutelier M , Blesneac I, Monteil A, Monin ML, Ando K, Mundwiller E, et al A recurrent mutation in *CACNA1G* alters Cav3.1 T-type calcium-channel conduction and causes autosomal-dominant cerebellar ataxia. Am J Hum Genet 2015;97:726–37. 26456284 10.1016/j.ajhg.2015.09.007 PMC4667105

[29] Li X , Zhou C, Cui L, Zhu L, Du H, Liu J, et al A case of a novel *CACNA1G* mutation from a Chinese family with SCA42: a case report and literature review. Medicine (Baltimore) 2018;97:e12148. 30200108 10.1097/MD.0000000000012148 PMC6133555

[30] Lin Z , Chen L, Chen X, Zhong Y, Yang Y, Xia W, et al Biological adaptations in the Arctic cervid, the reindeer (*Rangifer tarandus*). Science 2019;364:eaav6312. 31221829 10.1126/science.aav6312

[31] Trimmer JS , Cooperman SS, Tomiko SA, Zhou JY, Crean SM, Boyle MB, et al Primary structure and functional expression of a mammalian skeletal muscle sodium channel. Neuron 1989;3:33–49. 2559760 10.1016/0896-6273(89)90113-x

[32] Avila-Smirnow D , Vargas Leal CP, Beytía Reyes MLA, Cortés Zepeda R, Escobar RG, Kleinsteuber Saa K, et al Non-dystrophic myotonia Chilean cohort with predominance of the *SCN4A* Gly1306Glu variant. Neuromuscul Disord 2020;30:554–61. 32593548 10.1016/j.nmd.2020.04.006

[33] Jeong HN , Yi JS, Lee YH, Lee JH, Shin HY, Choi YC, et al Lower-extremity magnetic resonance imaging in patients with hyperkalemic periodic paralysis carrying the *SCN4A* mutation T704M: 30-month follow-up of seven patients. Neuromuscul Disord 2018;28:837–45. 30172468 10.1016/j.nmd.2018.06.008

[34] Berghold VM , Koko M, Berutti R, Plecko B. Case report: novel *SCN4* variant associated with a severe congenital myasthenic syndrome/myopathy phenotype. Front Pediatr 2022;10:944784. 36090556 10.3389/fped.2022.944784 PMC9462513

[35] Baxter LL , Watkins-Chow DE, Pavan WJ, Loftus SK. A curated gene list for expanding the horizons of pigmentation biology. Pigment Cell Melanoma Res 2019;32:348–58. 30339321 10.1111/pcmr.12743 PMC10413850

[36] Robbins LS , Nadeau JH, Johnson KR, Kelly MA, Roselli-Rehfuss L, Baack E, et al Pigmentation phenotypes of variant extension locus alleles result from point mutations that alter MSH receptor function. Cell 1993;72:827–34. 8458079 10.1016/0092-8674(93)90572-8

[37] Schiöth HB. The physiological role of melanocortin receptors. Vitam Horm 2001;63:195–232. 11358115 10.1016/s0083-6729(01)63007-3

[38] Gao D , Zhang S, Bi K, Sheng X, Dong C, Wu Z, et al Karst in south Guizhou, China. Guiyang: Guizhou People’s Publishing House; 1986.

[39] Matsubayashi H , Lagan P, Majalap N, Tangah J, Sukor JRA, Kitayama K. Importance of natural licks for the mammals in Bornean inland tropical rain forests. Ecol Res 2007;22:742–8.

[40] Matsuda I , Ancrenaz M, Akiyama Y, Tuuga A, Majalap N, Bernard H. Natural licks are required for large terrestrial mammals in a degraded riparian forest, Sabah, Borneo, Malaysia. Ecol Res 2015;30:191–5.

[41] Stunnenberg BC , LoRusso S, Arnold WD, Barohn RJ, Cannon SC, Fontaine B, et al Guidelines on clinical presentation and management of nondystrophic myotonias. Muscle Nerve 2020;62:430–44. 32270509 10.1002/mus.26887 PMC8117169

[42] Choi DW. Calcium-mediated neurotoxicity: relationship to specific channel types and role in ischemic damage. Trends Neurosci 1988;11:465–9. 2469166 10.1016/0166-2236(88)90200-7

[43] Zamponi GW , Striessnig J, Koschak A, Dolphin AC. The physiology, pathology, and pharmacology of voltage-gated calcium channels and their future therapeutic potential. Pharmacol Rev 2015;67:821–70. 26362469 10.1124/pr.114.009654 PMC4630564

[44] Vitko I , Chen Y, Arias JM, Shen Y, Wu XR, Perez-Reyes E. Functional characterization and neuronal modeling of the effects of childhood absence epilepsy variants of *CACNA1H*, a T-type calcium channel. J Neurosci 2005;25:4844–55. 15888660 10.1523/JNEUROSCI.0847-05.2005 PMC6724770

[45] Catterall WA. From ionic currents to molecular mechanisms: the structure and function of voltage-gated sodium channels. Neuron 2000;26:13–25. 10798388 10.1016/s0896-6273(00)81133-2

[46] Kuzmenkin A , Jurkat-Rott K, Lehmann-Horn F, Mitrovic N. Impaired slow inactivation due to a polymorphism and substitutions of Ser-906 in the II–III loop of the human Na_v_1.4 channel. Pflugers Arch 2003;447:71–7. 12898257 10.1007/s00424-003-1137-5

[47] Rubinstein M , Patowary A, Stanaway IB, McCord E, Nesbitt RR, Archer M, et al Association of rare missense variants in the second intracellular loop of Na_v_1.7 sodium channels with familial autism. Mol Psychiatry 2018;23:231–9. 27956748 10.1038/mp.2016.222 PMC5468514

[48] Wang L , Yin YL, Liu XZ, Shen P, Zheng YG, Lan XR, et al Current understanding of metal ions in the pathogenesis of Alzheimer’s disease. Transl Neurodegener 2020;9:10. 32266063 10.1186/s40035-020-00189-z PMC7119290

[49] Theophanides T , Anastassopoulou J. The effects of metal ion contaminants on the double stranded DNA helix and diseases. J Environ Sci Health A Tox Hazard Subst Environ Eng 2017;52:1030–40. 28758877 10.1080/10934529.2017.1328950

[50] Doil C , Mailand N, Bekker-Jensen S, Menard P, Larsen DH, Pepperkok R, et al RNF168 binds and amplifies ubiquitin conjugates on damaged chromosomes to allow accumulation of repair proteins. Cell 2009;136:435–46. 19203579 10.1016/j.cell.2008.12.041

[51] Thorslund T , Ripplinger A, Hoffmann S, Wild T, Uckelmann M, Villumsen B, et al Histone H1 couples initiation and amplification of ubiquitin signalling after DNA damage. Nature 2015;527:389–93. 26503038 10.1038/nature15401

[52] Wu CG , Zheng A, Jiang L, Rowse M, Stanevich V, Chen H, et al Methylation-regulated decommissioning of multimeric PP2A complexes. Nat Commun 2017;8:2272. 29273778 10.1038/s41467-017-02405-3 PMC5741625

[53] Arshad M , Abdul Hamid N, Chan MC, Ismail F, Tan GC, Pezzella F, et al NUB1 and FAT10 proteins as potential novel biomarkers in cancer: a translational perspective. Cells 2021;10:2176. 34571823 10.3390/cells10092176 PMC8468723

[54] Stevens M , Merilaita S. Animal camouflage: mechanisms and function. New York: Cambridge University Press; 2011.

[55] Caro T , Mallarino R. Coloration in mammals. Trends Ecol Evol 2020;35:357–66. 31980234 10.1016/j.tree.2019.12.008 PMC10754262

[56] Benned-Jensen T , Mokrosinski J, Rosenkilde MM. The E92K melanocortin 1 receptor mutant induces cAMP production and arrestin recruitment but not ERK activity indicating biased constitutive signaling. PLoS One 2011;6:e24644. 21931793 10.1371/journal.pone.0024644 PMC3172247

[57] Takeuchi S , Suzuki H, Yabuuchi M, Takahashi S. A possible involvement of melanocortin 1-receptor in regulating feather color pigmentation in the chicken. Biochim Biophys Acta 1996;1308:164–8. 8764834 10.1016/0167-4781(96)00100-5

[58] Theron E , Hawkins K, Bermingham E, Ricklefs RE, Mundy NI. The molecular basis of an avian plumage polymorphism in the wild: a melanocortin-1-receptor point mutation is perfectly associated with the melanic plumage morph of the bananaquit, *Coereba flaveola*. Curr Biol 2001;11:550–7. 11369199 10.1016/s0960-9822(01)00158-0

[59] Hassani A , Azapagic A, Shokri N. Global predictions of primary soil salinization under changing climate in the 21st century. Nat Commun 2021;12:6663. 34795219 10.1038/s41467-021-26907-3 PMC8602669

[60] Cheng H , Concepcion GT, Feng X, Zhang H, Li H. Haplotype-resolved *de novo* assembly using phased assembly graphs with hifiasm. Nat Methods 2021;18:170–5. 33526886 10.1038/s41592-020-01056-5 PMC7961889

[61] Dudchenko O , Batra SS, Omer AD, Nyquist SK, Hoeger M, Durand NC, et al *De novo* assembly of the *Aedes aegypti* genome using Hi-C yields chromosome-length scaffolds. Science 2017;356:92–5. 28336562 10.1126/science.aal3327 PMC5635820

[62] Harris RS. Improved pairwise alignment of genomic DNA. A Ph.D. thesis. The Pennsylvania State University; 2007.

[63] Kurtz S , Phillippy A, Delcher AL, Smoot M, Shumway M, Antonescu C, et al Versatile and open software for comparing large genomes. Genome Biol 2004;5:R12. 14759262 10.1186/gb-2004-5-2-r12 PMC395750

[64] Krzywinski MI , Schein JE, Birol I, Connors J, Gascoyne R, Horsman D, et al Circos: an information aesthetic for comparative genomics. Genome Res 2009;19:1639–45. 19541911 10.1101/gr.092759.109 PMC2752132

[65] Gurevich A , Saveliev V, Vyahhi N, Tesler G. QUAST: quality assessment tool for genome assemblies. Bioinformatics 2013;29:1072–5. 23422339 10.1093/bioinformatics/btt086 PMC3624806

[66] Cantarel BL , Korf I, Robb SMC, Parra G, Ross E, Moore B, et al MAKER: an easy-to-use annotation pipeline designed for emerging model organism genomes. Genome Res 2008;18:188–96. 18025269 10.1101/gr.6743907 PMC2134774

[67] Cantalapiedra CP , Hernández-Plaza A, Letunic I, Bork P, Huerta-Cepas J. EggNOG-mapper v2: functional annotation, orthology assignments, and domain prediction at the metagenomic scale. Mol Biol Evol 2021;38:5825–9. 34597405 10.1093/molbev/msab293 PMC8662613

[68] Meng G , Li Y, Yang C, Liu S. MitoZ: a toolkit for animal mitochondrial genome assembly, annotation and visualization. Nucleic Acids Res 2019;47:e63. 30864657 10.1093/nar/gkz173 PMC6582343

[69] Altschul SF , Gish W, Miller W, Myers EW, Lipman DJ. Basic local alignment search tool. J Mol Biol 1990;215:403–10. 2231712 10.1016/S0022-2836(05)80360-2

[70] Katoh K , Standley DM. MAFFT multiple sequence alignment software version 7: improvements in performance and usability. Mol Biol Evol 2013;30:772–80. 23329690 10.1093/molbev/mst010 PMC3603318

[71] Löytynoja A. Phylogeny-aware alignment with PRANK. Methods Mol Biol 2014;1079:155–70. 24170401 10.1007/978-1-62703-646-7_10

[72] De Bie T , Cristianini N, Demuth JP, Hahn MW. CAFE: a computational tool for the study of gene family evolution. Bioinformatics 2006;22:1269–71. 16543274 10.1093/bioinformatics/btl097

[73] Danecek P , Bonfield JK, Liddle J, Marshall J, Ohan V, Pollard MO, et al, Twelve years of SAMtools and BCFtools. Gigascience 2021;10:giab008. 33590861 10.1093/gigascience/giab008 PMC7931819

[74] Stamatakis A. RAxML version 8: a tool for phylogenetic analysis and post-analysis of large phylogenies. Bioinformatics 2014;30:1312–3. 24451623 10.1093/bioinformatics/btu033 PMC3998144

[75] Minh BQ , Schmidt HA, Chernomor O, Schrempf D, Woodhams MD, von Haeseler A, et al IQ-TREE 2: new models and efficient methods for phylogenetic inference in the genomic era. Mol Biol Evol 2020;37:1530–4. 32011700 10.1093/molbev/msaa015 PMC7182206

[76] de Vries D , Beck RMD. Twenty-five well-justified fossil calibrations for primate divergences. Palaeontol Electronica 2023;26:a8.

[77] Bouckaert R , Vaughan TG, Barido-Sottani J, Duchêne S, Fourment M, Gavryushkina A, et al BEAST 2.5: an advanced software platform for Bayesian evolutionary analysis. PLoS Comput Biol 2019;15:e1006650. 30958812 10.1371/journal.pcbi.1006650 PMC6472827

[78] Rambaut A , Drummond AJ, Xie D, Baele G, Suchard MA. Posterior summarization in Bayesian phylogenetics using Tracer 1.7. Syst Biol 2018;67:901–4. 29718447 10.1093/sysbio/syy032 PMC6101584

[79] Harrison PW , Jordan GE, Montgomery SH. SWAMP: Sliding Window Alignment Masker for PAML. Evol Bioinform Online 2014;10:197–204. 25525323 10.4137/EBO.S18193 PMC4251194

[80] Zhou Y , Zhou B, Pache L, Chang M, Khodabakhshi AH, Tanaseichuk O, et al Metascape provides a biologist-oriented resource for the analysis of systems-level datasets. Nat Commun 2019;10:1523. 30944313 10.1038/s41467-019-09234-6 PMC6447622

[81] Ochoa D , Pazos F. Practical aspects of protein co-evolution. Front Cell Dev Biol 2014;2:14. 25364721 10.3389/fcell.2014.00014 PMC4207036

[82] Pan Y , Chai X, Gao Q, Zhou L, Zhang S, Li L, et al Dynamic interactions of plant CNGC subunits and calmodulins drive oscillatory Ca^2+^ channel activities. Dev Cell 2019;48:710–25.e5. 30713075 10.1016/j.devcel.2018.12.025

[83] Trachsel DS , Tejada MA, Groesfjeld Christensen V, Pedersen PJ, Kanters JK, Buhl R, et al Effects of trimethoprim–sulfadiazine and detomidine on the function of equine K_v_11.1 channels in a two-electrode voltage-clamp (TEVC) oocyte model. J Vet Pharmacol Ther 2018;41:536–45. 29566261 10.1111/jvp.12502

[84] Yan X , Terai Y, Widayati KA, Itoigawa A, Purba LHPS, Fahri F, et al Functional divergence of the pigmentation gene melanocortin-1 receptor (MC1R) in six endemic *Macaca* species on Sulawesi Island. Sci Rep 2022;12:7593. 35534524 10.1038/s41598-022-11681-z PMC9085793

[85] Chen T, Chen X, Zhang S, Zhu J, Tang B, Wang A, et al. The Genome Sequence Archive Family: toward explosive data growth and diverse data types. Genomics Proteomics Bioinformatics 2021;19:578–83. 10.1016/j.gpb.2021.08.001 PMC9039563 34400360

[86] Chen M, , Ma Y, , Wu S, Zheng X, Kang H, Sang J, et al Genome Warehouse: a public repository housing genome-scale data. Genomics Proteomics Bioinformatics. 2021;19:584–9. 34175476 10.1016/j.gpb.2021.04.001 PMC9039550

