# Supplementary material for: Living on the Rocks: Genomic Analysis of Limestone Langurs Provides Novel Insights into the Adaptive Evolution in Extreme Karst Environments
Source: Genomics Proteomics Bioinformatics. 2025 Feb 11;23(1):qzaf007. doi: 10.1093/gpbjnl/qzaf007 (PMC12231542; doi:10.1093/gpbjnl/qzaf007)
Supplement: qzaf007_Supplementary_Data [file qzaf007_supplementary_data.zip › Supplementary material captions.docx]

**Supplementary material**

**Figure S1 Chromatin interaction between 23 langur pseudo-chromosomes**

Each heat map shows a normalized contact matrix, with strong contacts in red.

**Figure S2 Intergenomic synteny**

Synteny demonstration between langur genome (Tfra_5.0) and human genome ([GRCh38.p14](https://www.ncbi.nlm.nih.gov/datasets/genome/GCF_000001405.40/)) (**A**) and between langur genome (Tfra_5.0) and macaque genome ([Mmul_10](https://www.ncbi.nlm.nih.gov/datasets/genome/GCF_003339765.1/)) (**B**).

**Figure S3 Deamination patterns at the edges of the next generation sequencing reads estimated by mapDamage2.0 for each of the 48 samples**

The vertical axis represents different samples. The horizontal axis shows the C to T deamination frequency at the 5' end (in red) and the G to A rate at the 3' end (in blue).

**Figure S4 Ortholog delineation among the protein-coding gene family repertoires of *T. francoisi***

The single-copy orthologs are the only one copy per species, the multiple-copy orthologs are the numerous copies per species, the unique paralogs are found only in a single species, the unclustered genes have no homology with others, the other orthologs are the rest.

**Figure S5 Gene family evolution**

**A.** Numbers of contraction and expansion gene family during the evolution of ten species. **B.** KEGG pathways analysis of the expanded gene families in *T. francoisi*. MRCA, most recent common ancestor; KEGG, Kyoto Encyclopedia of Genes and Genomes.

**Figure S6** **Phylogeny and divergence times of *Trachypithecus* spp.**

Phylogeny and divergence times of *Trachypithecus* spp. based on single-copy orthologs of nuclear genes (**A**) and mitochrodrial genomes (**B**). Red dots denote the utilized calibration points. Node bars refer to 95% HPD intervals of estimated divergence times. HPD, highest posterior densities.

**Figure S7 Phylogenetic analysis of *Trachypithecus* spp.**

Phylogenetic analysis of *Trachypithecus* spp. based on the CDS on the X chromosome (**A**) and Y chromosome (**B**).

**Figure S8 Comparison of sequence integrity**

Comparison of the completeness of α1 subunit of the ten calcium ion channels and ten sodium ion channels in GRCh38 (human), Mmul_10 (rhesus macaque), Tfra_5.0, and Tfra_2.0.

**Figure S9 Concentrations of minerals in water from natural limestone ponds and blood samples of Francois’s langurs**

**A.** Concentrations (mean ± SD) of several mineral ion and the pH value in the water samples in natural ponds and nearby river (controls), respectively. **B.** Comparative statistics of mineral ion concentration (mean ± SD) in the blood samples from *T. francoisi*, *M. fascicularis*, *M. mulatta*, and *H. sapiens* (Wilcoxon two-sided test; *, *P* < 0.05; **, *P* < 0.01; ***, *P* < 0.001; ****, *P* < 0.0001). SD, standard deviation.

**Figure S10 Relative accumulation of mutants and wild-type of CACNA1G and SCN4A in HEK-293T cells**

**A.** Representative immunoblots from independent transfections performed as described in methods. For quantification, immunoreactivities of individual mutants and wild-type of CACNA1G were normalized to β-actin as loading control. **B.** Quantification of expression levels of mutants of CACNA1G normalized to the respective wild-type of CACNA1G [mean ± SD (% of CACNA1G): A8T: 1.06 ± 0.39; Q453R: 1.11 ± 0.34; S2370A: 0.95 ± 0.21; A8T-Q453R-S2370A: 1.20 ± 0.42; n = 3 for each condition, not significant, Wilcoxon test]. **C.** Representative immunoblots from independent transfections performed as described in methods. For quantification, immunoreactivities of individual mutant and wild-type of SCN4A were normalized to β-actin as loading control. **D.** Quantification of expression levels of mutant of SCN4A normalized to the respective wild-type of SCN4A [mean ± SD (% of SCN4A): G1824E: 1.10 ± 0.24; n = 3 for each condition, not significant, Wilcoxon test].

**Table S1 Summary of the 23 chromosome-scale pseudo-molecules (pseudo-chromosomes) in the Tfra_5.0 langur assembly**

**Table S2 Annotation of repeat sequences in the langur genome**

**Table S3** **Sample information and the sequencing statistics of the genome resequencing of 48 langurs**

**Table S4 List of 58 rapidly evolving genes in *T. francoisi***

**Table S5 List of 37 positively selected genes in *T. francoisi* and the** **amino acid substitutions**

**Table S6 Fixed amino acid substitutions in calcium channel α1 subunit, sodium channel α1 subunit, and potassium channel genes of limestone langurs and the allele frequencies in human gnomAD database**

**Table S7 Amino acid substitutions in calcium channels α1 subunit, sodium channels α1 subunit, and potassium channels for seven limestone langurs in comparison to 23 other primate species**

**Table S8** **124 pelage color genes with fixed limestone langur-specific amino acid substitutions in all seven limestone langur species**
