# Supplementary figures and images for: Living on the Rocks: Genomic Analysis of Limestone Langurs Provides Novel Insights into the Adaptive Evolution in Extreme Karst Environments
Source: Genomics Proteomics Bioinformatics. 2025 Feb 11;23(1):qzaf007. doi: 10.1093/gpbjnl/qzaf007 (PMC12231542; doi:10.1093/gpbjnl/qzaf007)

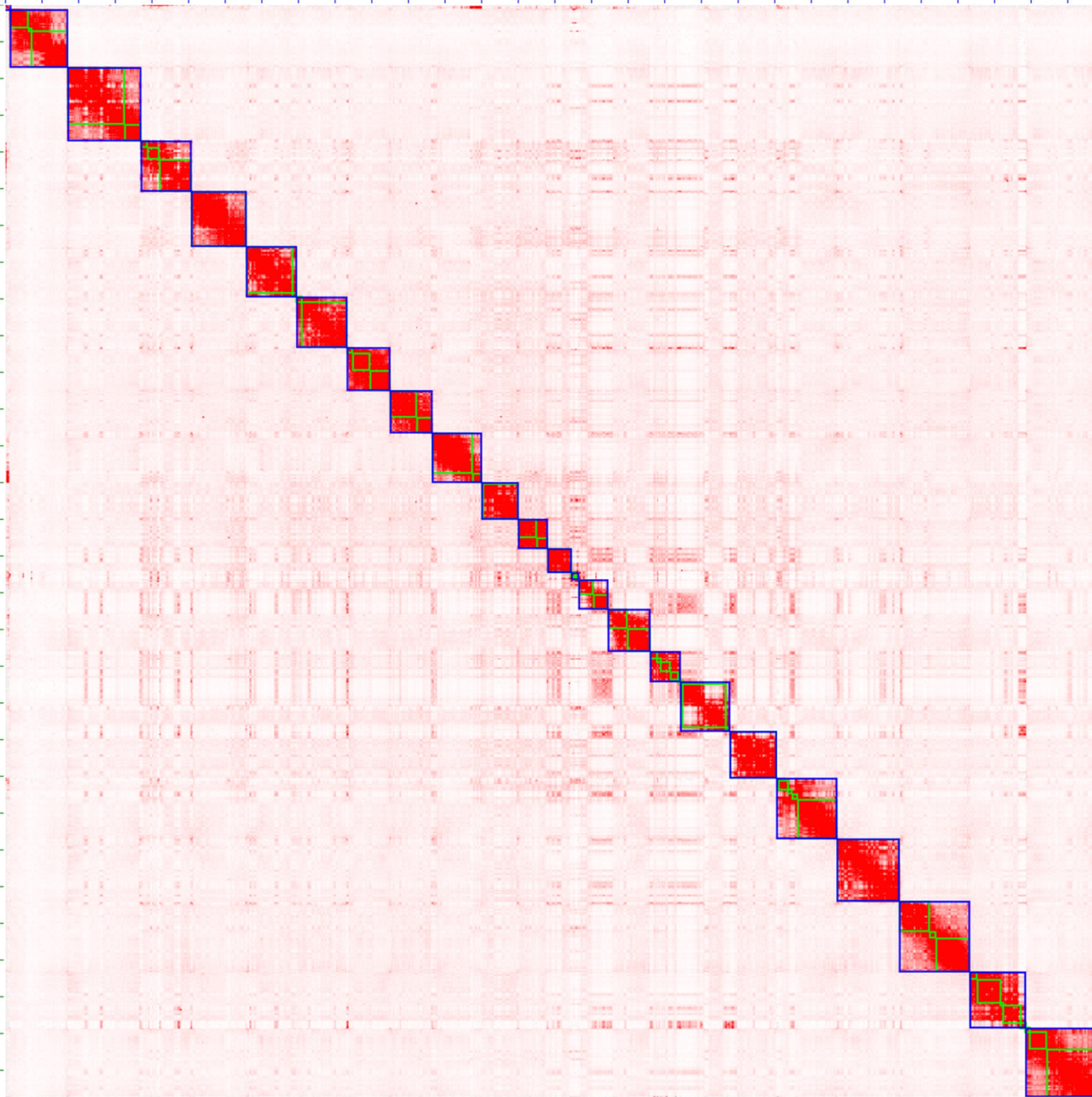

Supplement: qzaf007_Supplementary_Data [file qzaf007_supplementary_data.zip › FigS1.pdf]

A

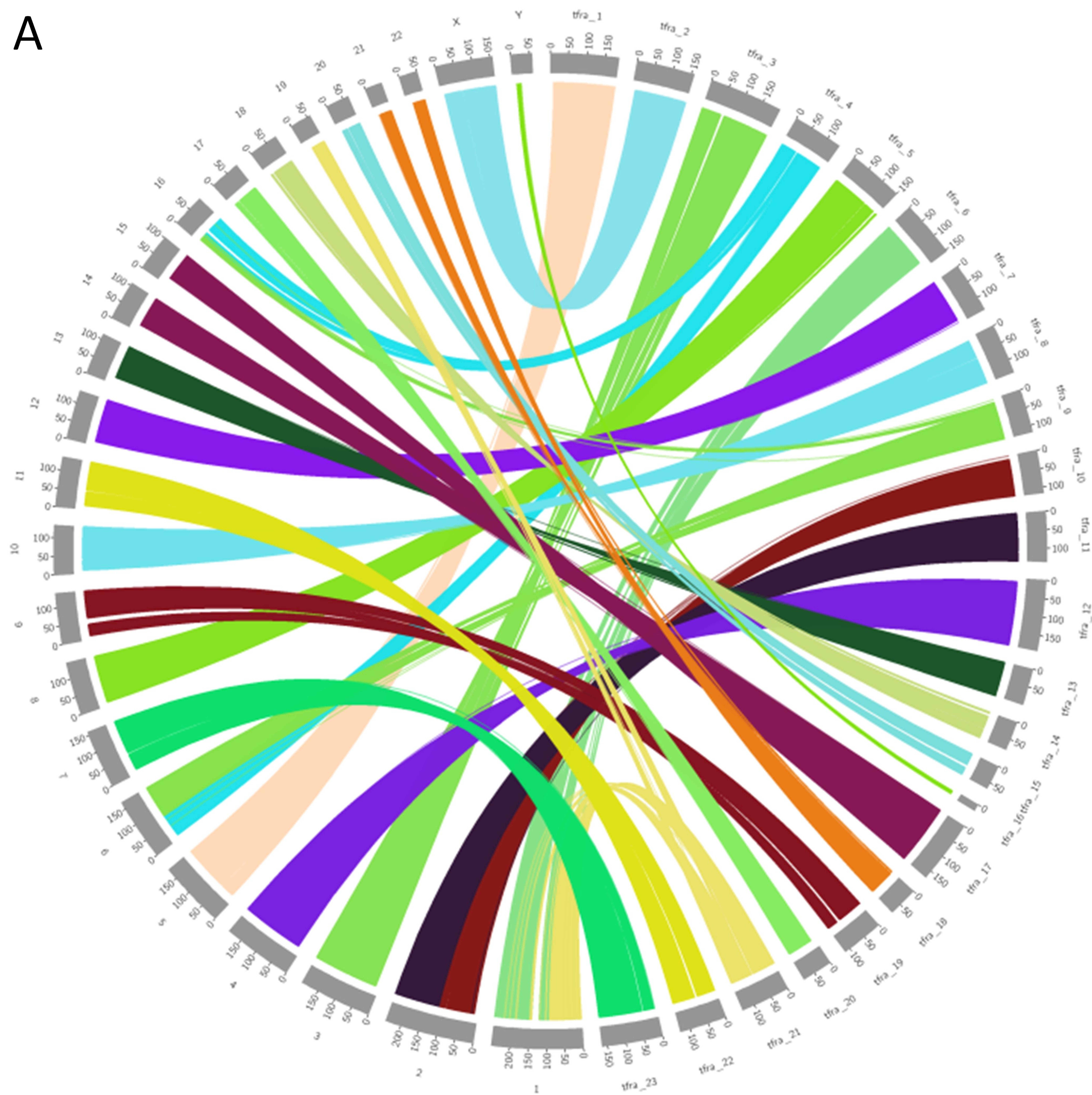

B

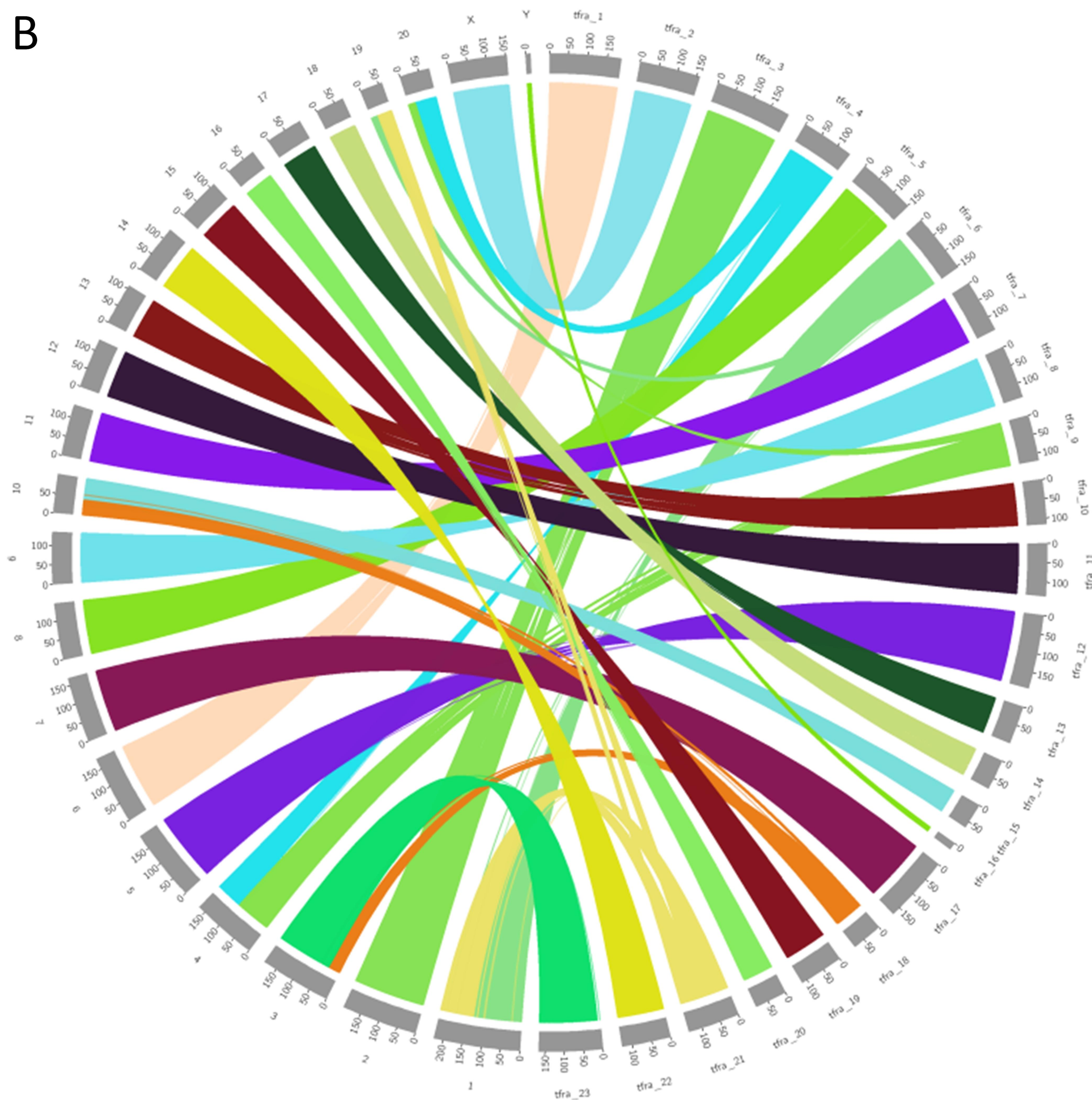

Supplement: qzaf007_Supplementary_Data [file qzaf007_supplementary_data.zip › FigS2.pdf]
