# Supplementary material for: Living on the Rocks: Genomic Analysis of Limestone Langurs Provides Novel Insights into the Adaptive Evolution in Extreme Karst Environments
Source: Genomics Proteomics Bioinformatics. 2025 Feb 11;23(1):qzaf007. doi: 10.1093/gpbjnl/qzaf007 (PMC12231542; doi:10.1093/gpbjnl/qzaf007)
Supplement: qzaf007_Supplementary_Data [file FigS10.pdf › FigS10.pdf]

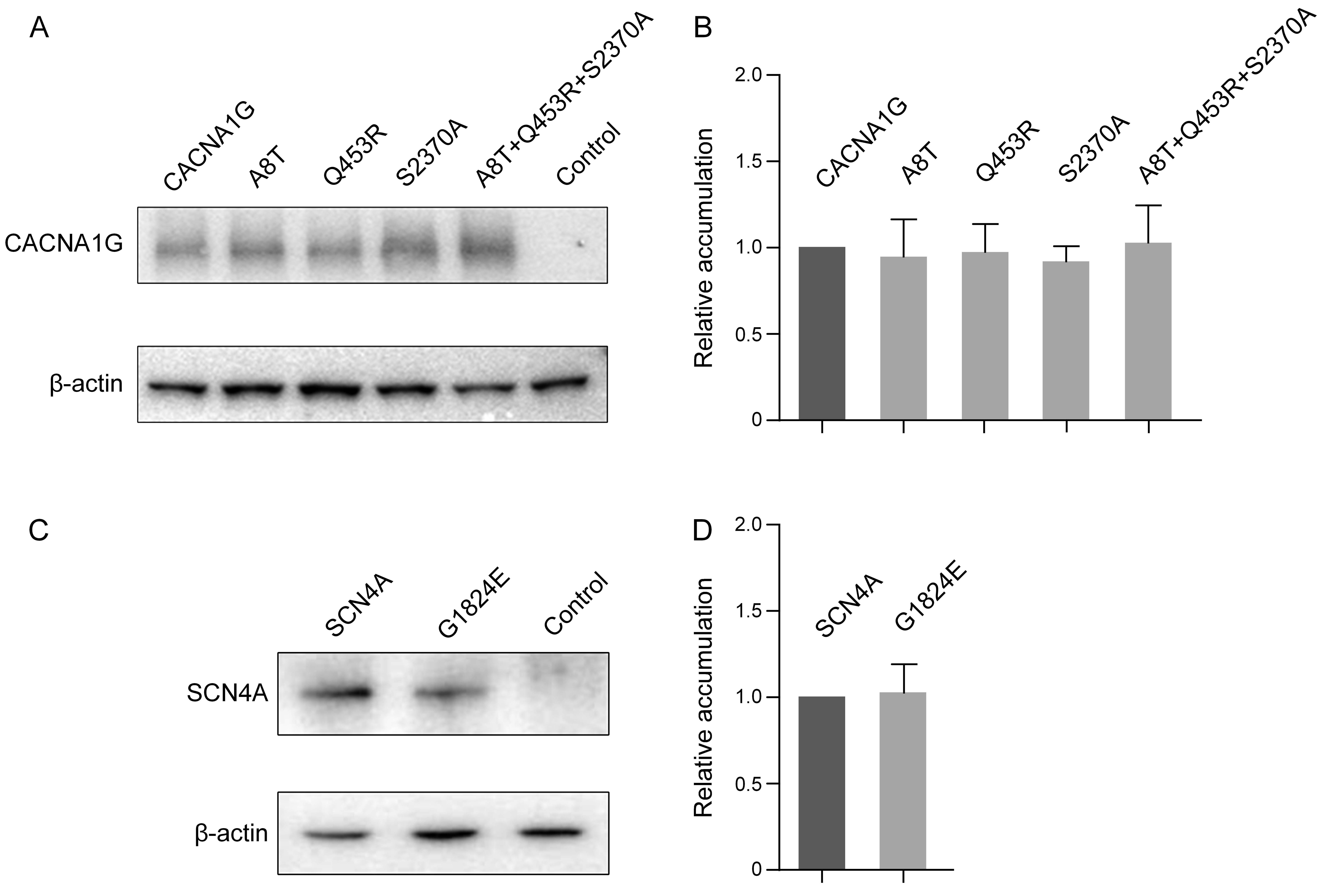

A
CACNA1G
A8T
Q453R
S2370A
A8T+Q453R+S2370A
Control
CACNA1G
β-actin
B
Relative accumulation
2.0
1.5
1.0
0.5
0
CACNA1G
A8T
Q453R
S2370A
A8T+Q453R+S2370A
C
SCN4A
G1824E
Control
SCN4A
β-actin
D
Relative accumulation
2.0
1.5
1.0
0.5
0
SCN4A
G1824E
