# Supplementary material for: Living on the Rocks: Genomic Analysis of Limestone Langurs Provides Novel Insights into the Adaptive Evolution in Extreme Karst Environments
Source: Genomics Proteomics Bioinformatics. 2025 Feb 11;23(1):qzaf007. doi: 10.1093/gpbjnl/qzaf007 (PMC12231542; doi:10.1093/gpbjnl/qzaf007)
Supplement: qzaf007_Supplementary_Data [file FigS3.pdf › FigS3.pdf]

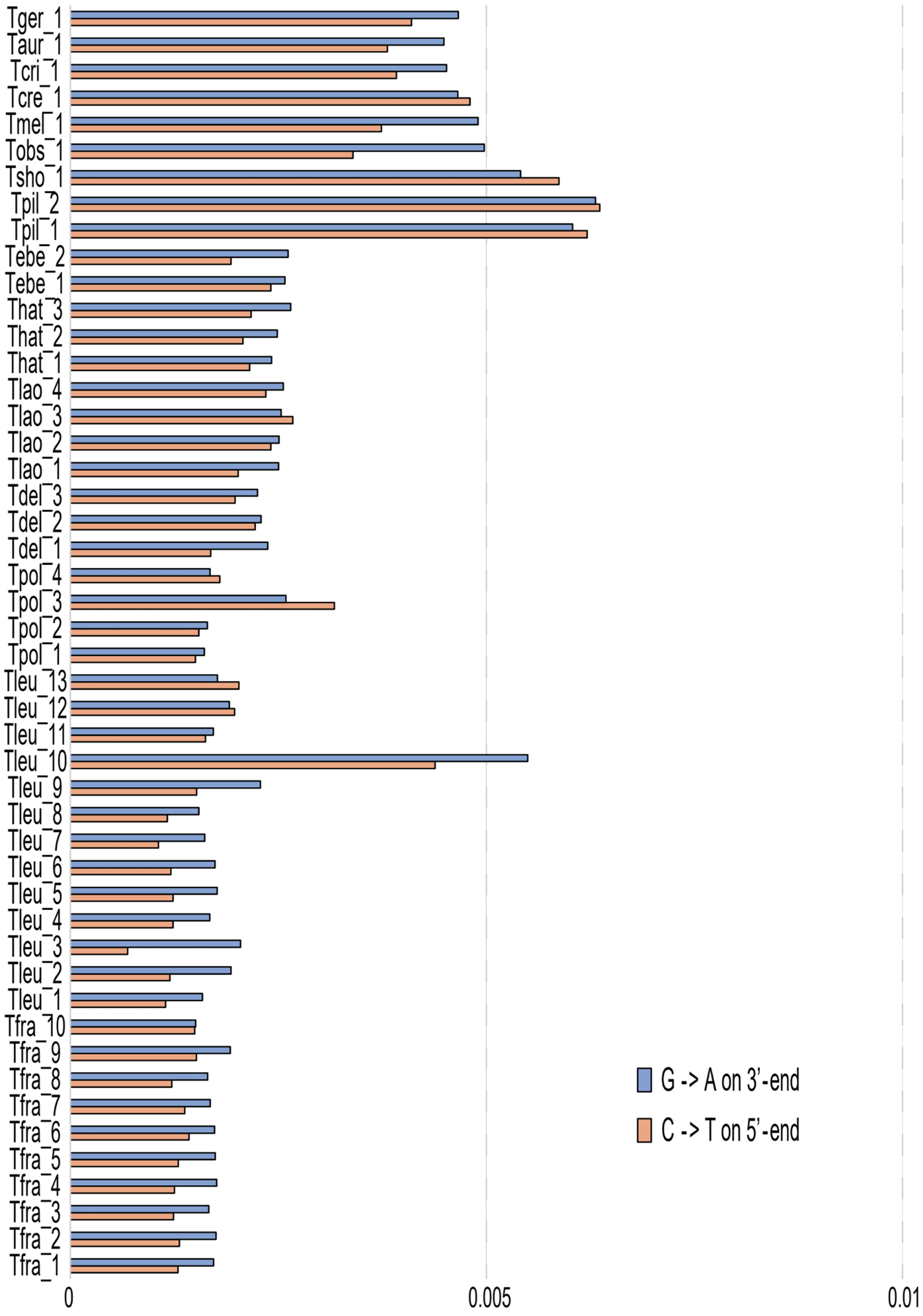

Tger_1
Taur_1
Tcri_1
Tcre_1
Tmel_1
Tobs_1
Tsho_1
Tpil_2
Tpil_1
Tebe_2
Tebe_1
That_3
That_2
That_1
Tlao_4
Tlao_3
Tlao_2
Tlao_1
Tdel_3
Tdel_2
Tdel_1
Tpol_4
Tpol_3
Tpol_2
Tpol_1
Tleu_13
Tleu_12
Tleu_11
Tleu_10
Tleu_9
Tleu_8
Tleu_7
Tleu_6
Tleu_5
Tleu_4
Tleu_3
Tleu_2
Tleu_1
Tfra_10
Tfra_9
Tfra_8
Tfra_7
Tfra_6
Tfra_5
Tfra_4
Tfra_3
Tfra_2
Tfra_1
0
0.005
0.01
G -> A on 3'-end
C -> T on 5'-end
