# Supplementary material for: Living on the Rocks: Genomic Analysis of Limestone Langurs Provides Novel Insights into the Adaptive Evolution in Extreme Karst Environments
Source: Genomics Proteomics Bioinformatics. 2025 Feb 11;23(1):qzaf007. doi: 10.1093/gpbjnl/qzaf007 (PMC12231542; doi:10.1093/gpbjnl/qzaf007)
Supplement: qzaf007_Supplementary_Data [file FigS4.pdf › FigS4.pdf]

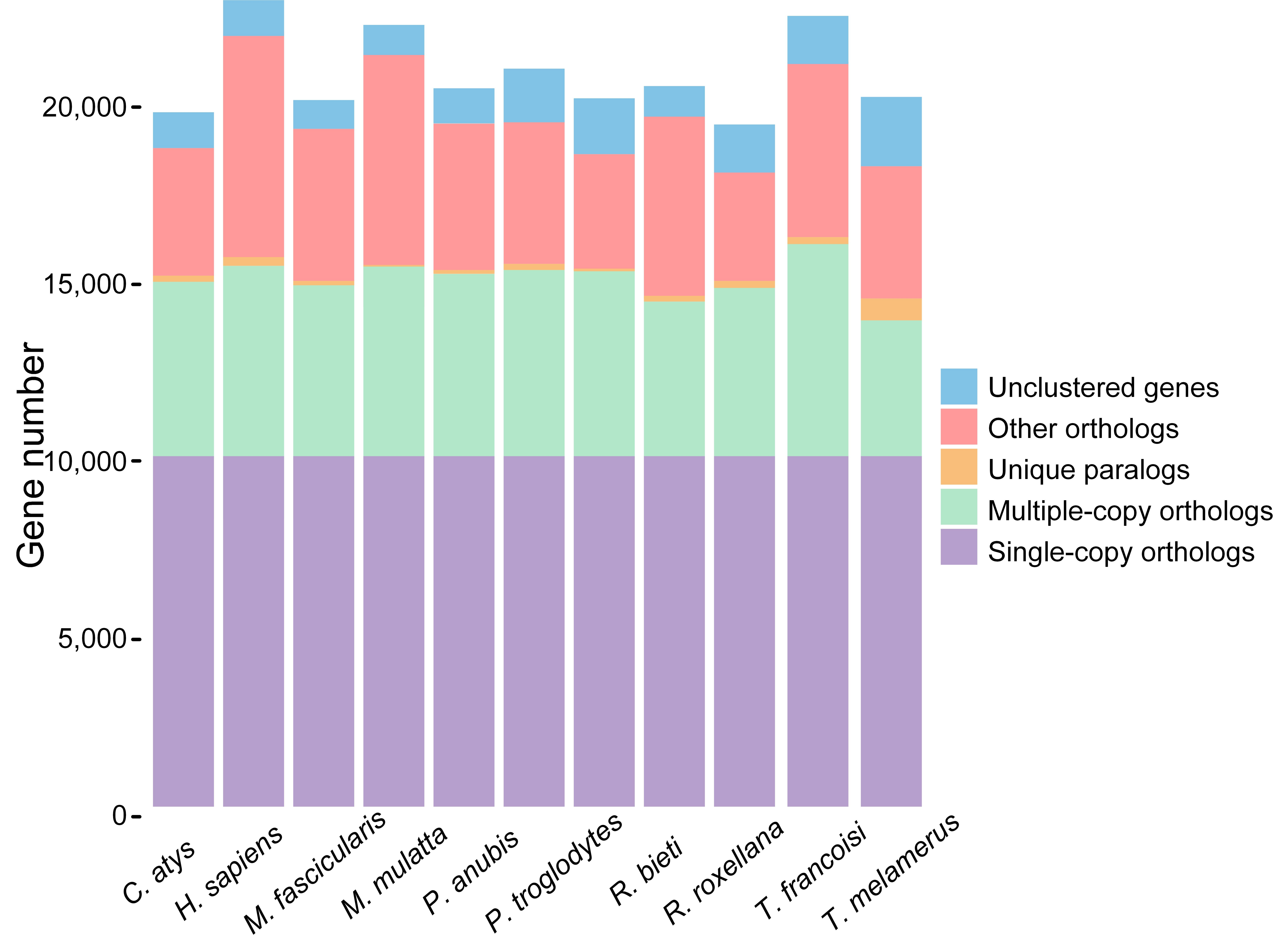

Gene number
0
5,000
10,000
15,000
20,000
C. atys
H. sapiens
M. fascicularis
M. mulatta
P. anubis
P. troglodytes
R. bieti
R. roxellana
T. francoisi
T. melamerus
Unclustered genes
Other orthologs
Unique paralogs
Multiple-copy orthologs
Single-copy orthologs
