# Supplementary material for: Living on the Rocks: Genomic Analysis of Limestone Langurs Provides Novel Insights into the Adaptive Evolution in Extreme Karst Environments
Source: Genomics Proteomics Bioinformatics. 2025 Feb 11;23(1):qzaf007. doi: 10.1093/gpbjnl/qzaf007 (PMC12231542; doi:10.1093/gpbjnl/qzaf007)
Supplement: qzaf007_Supplementary_Data [file FigS5.pdf › FigS5.pdf]

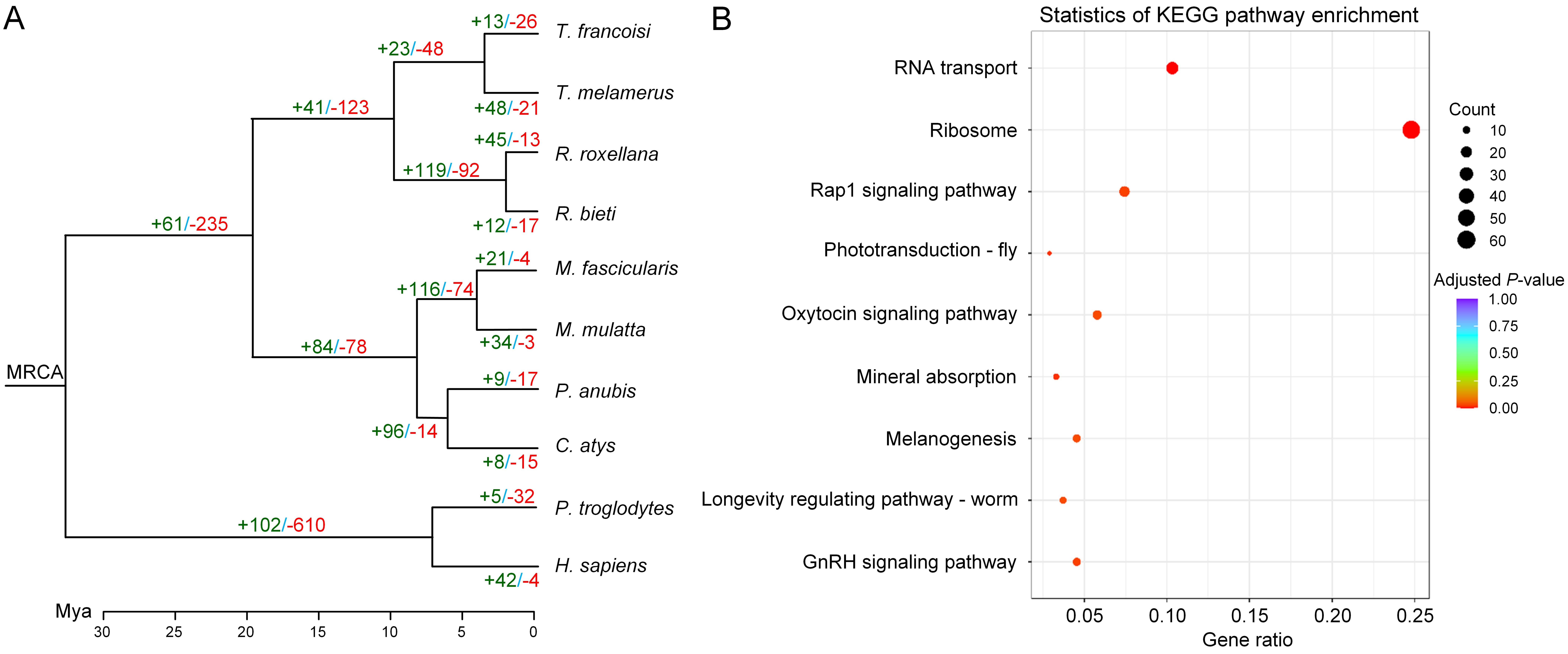
A
+13/-26
T. francoisi
+23/-48
T. melamerus
+48/-21
+41/-123
+45/-13
R. roxellana
+119/-92
R. bieti
+12/-17
+61/-235
+21/-4
M. fascicularis
+116/-74
M. mulatta
+34/-3
+84/-78
MRCA
+9/-17
P. anubis
+96/-14
C. atys
+8/-15
+5/-32
P. troglodytes
+102/-610
H. sapiens
+42/-4
Mya
30
25
20
15
10
5
0
B
Statistics of KEGG pathway enrichment
RNA transport
Ribosome
Rap1 signaling pathway
Phototransduction - fly
Oxytocin signaling pathway
Mineral absorption
Melanogenesis
Longevity regulating pathway - worm
GnRH signaling pathway
0.05
0.10
0.15
0.20
0.25
Gene ratio
Count
10
20
30
40
50
60
Adjusted P-value
1.00
0.75
0.50
0.25
0.00
