# Supplementary material for: Living on the Rocks: Genomic Analysis of Limestone Langurs Provides Novel Insights into the Adaptive Evolution in Extreme Karst Environments
Source: Genomics Proteomics Bioinformatics. 2025 Feb 11;23(1):qzaf007. doi: 10.1093/gpbjnl/qzaf007 (PMC12231542; doi:10.1093/gpbjnl/qzaf007)
Supplement: qzaf007_Supplementary_Data [file FigS6.pdf › FigS6.pdf]

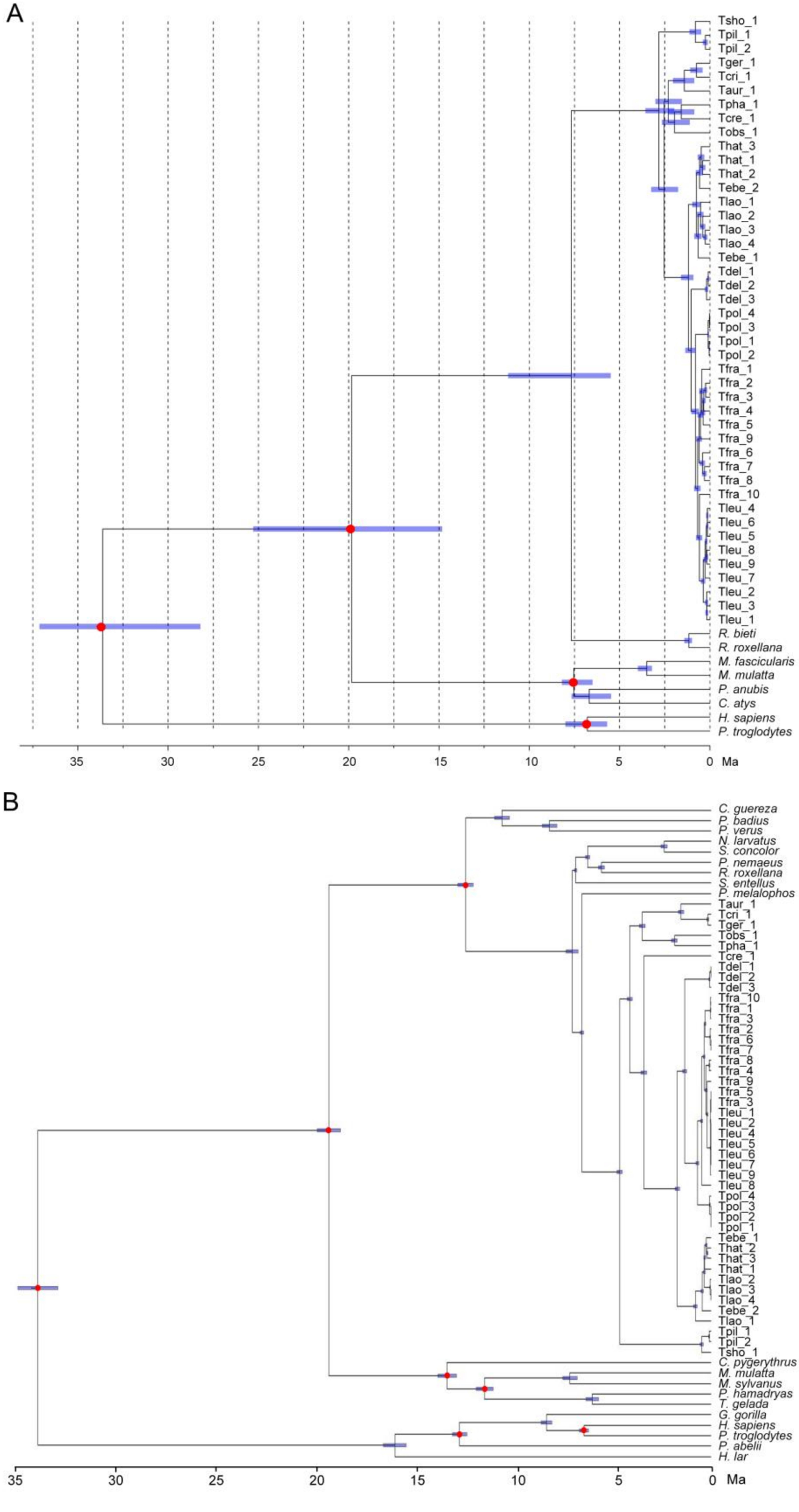

A
Tsho_1
Tpil_1
Tpil_2
Tger_1
Tcri_1
Taur_1
Tpha_1
Tcre_1
Tobs_1
That_3
That_1
That_2
Tebe_2
Tlao_1
Tlao_2
Tlao_3
Tlao_4
Tebe_1
Tdel_1
Tdel_2
Tdel_3
Tpol_4
Tpol_3
Tpol_1
Tpol_2
Tfra_1
Tfra_2
Tfra_3
Tfra_4
Tfra_5
Tfra_9
Tfra_6
Tfra_7
Tfra_8
Tfra_10
Tleu_4
Tleu_6
Tleu_5
Tleu_8
Tleu_9
Tleu_7
Tleu_2
Tleu_3
Tleu_1
R. bieti
R. roxellana
M. fascicularis
M. mulatta
P. anubis
C. atys
H. sapiens
P. troglodytes
35 30 25 20 15 10 5 0 Ma
B
C. guereza
P. badius
P. verus
N. larvatus
S. concolor
P. nemaeus
R. roxellana
S. entellus
P. melalophos
Taur_1
Tcri_1
Tger_1
Tobs_1
Tpha_1
Tcre_1
Tdel_1
Tdel_2
Tdel_3
Tfra_10
Tfra_1
Tfra_3
Tfra_2
Tfra_6
Tfra_7
Tfra_8
Tfra_4
Tfra_9
Tfra_5
Tfra_3
Tleu_1
Tleu_2
Tleu_4
Tleu_5
Tleu_6
Tleu_7
Tleu_9
Tleu_8
Tpol_4
Tpol_3
Tpol_2
Tpol_1
Tebe_1
That_2
That_3
That_1
Tlao_2
Tlao_3
Tlao_4
Tebe_2
Tlao_1
Tpil_1
Tpil_2
Tsho_1
C. pygerythrus
M. mulatta
M. sylvanus
P. hamadryas
T. gelada
G. gorilla
H. sapiens
P. troglodytes
P. abelii
H. lar
35 30 25 20 15 10 5 0 Ma
