# Supplementary material for: Living on the Rocks: Genomic Analysis of Limestone Langurs Provides Novel Insights into the Adaptive Evolution in Extreme Karst Environments
Source: Genomics Proteomics Bioinformatics. 2025 Feb 11;23(1):qzaf007. doi: 10.1093/gpbjnl/qzaf007 (PMC12231542; doi:10.1093/gpbjnl/qzaf007)
Supplement: qzaf007_Supplementary_Data [file FigS7.pdf › FigS7.pdf]

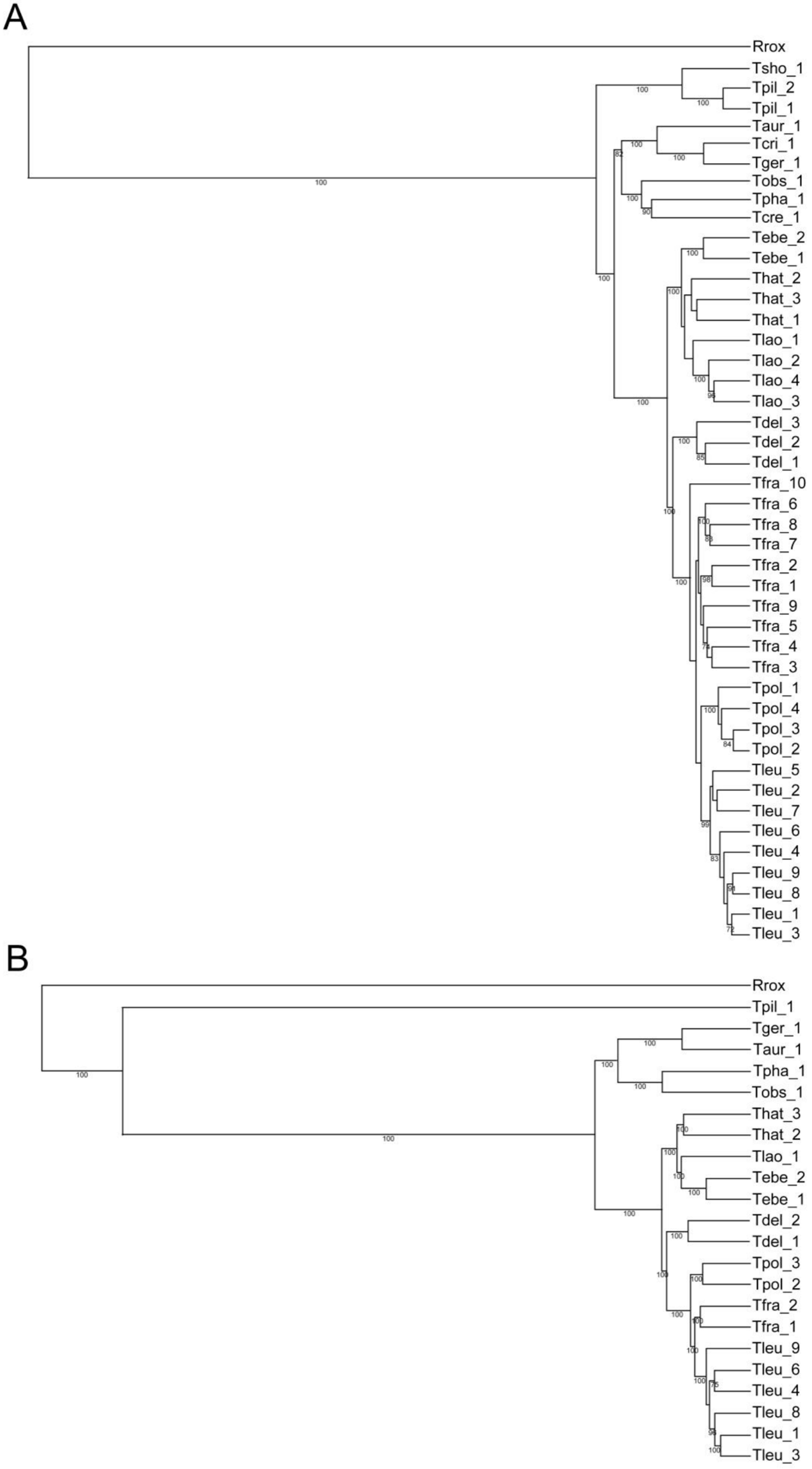
A
Rrox
Tsho_1
Tpil_2
Tpil_1
Taur_1
Tcri_1
Tger_1
Tobs_1
Tpha_1
Tcre_1
Tebe_2
Tebe_1
That_2
That_3
That_1
Tlao_1
Tlao_2
Tlao_4
Tlao_3
Tdel_3
Tdel_2
Tdel_1
Tfra_10
Tfra_6
Tfra_8
Tfra_7
Tfra_2
Tfra_1
Tfra_9
Tfra_5
Tfra_4
Tfra_3
Tpol_1
Tpol_4
Tpol_3
Tpol_2
Tleu_5
Tleu_2
Tleu_7
Tleu_6
Tleu_4
Tleu_9
Tleu_8
Tleu_1
Tleu_3
B
Rrox
Tpil_1
Tger_1
Taur_1
Tpha_1
Tobs_1
That_3
That_2
Tlao_1
Tebe_2
Tebe_1
Tdel_2
Tdel_1
Tpol_3
Tpol_2
Tfra_2
Tfra_1
Tleu_9
Tleu_6
Tleu_4
Tleu_8
Tleu_1
Tleu_3
