# Supplementary material for: Living on the Rocks: Genomic Analysis of Limestone Langurs Provides Novel Insights into the Adaptive Evolution in Extreme Karst Environments
Source: Genomics Proteomics Bioinformatics. 2025 Feb 11;23(1):qzaf007. doi: 10.1093/gpbjnl/qzaf007 (PMC12231542; doi:10.1093/gpbjnl/qzaf007)
Supplement: qzaf007_Supplementary_Data [file FigS8.pdf › FigS8.pdf]

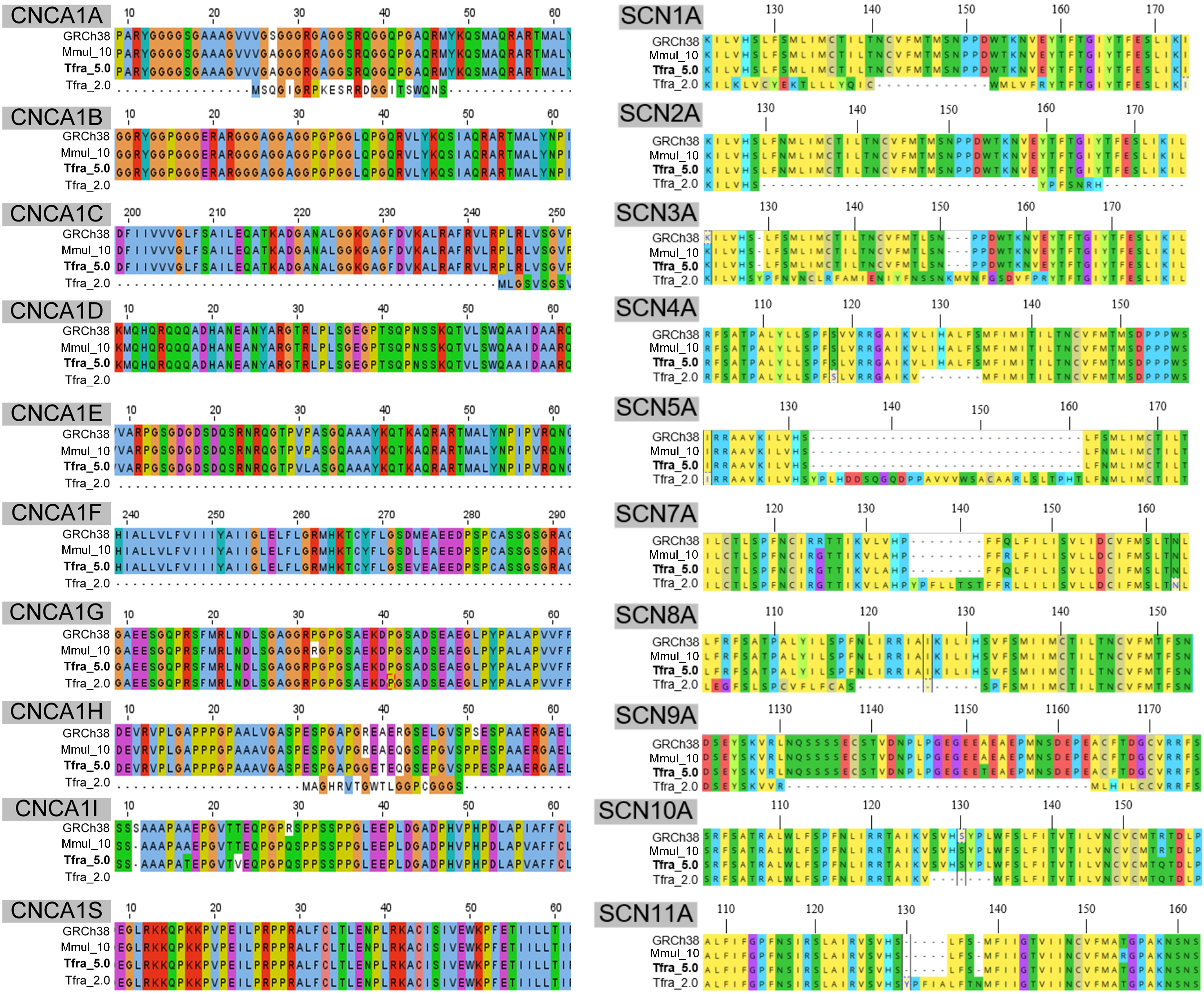
CNCA1A
CNCA1B
CNCA1C
CNCA1D
CNCA1E
CNCA1F
CNCA1G
CNCA1H
CNCA1I
CNCA1S
SCN1A
SCN2A
SCN3A
SCN4A
SCN5A
SCN7A
SCN8A
SCN9A
SCN10A
SCN11A
GRCh38
Mmul_10
Tfra_5.0
Tfra_2.0
