# Supplementary material for: Living on the Rocks: Genomic Analysis of Limestone Langurs Provides Novel Insights into the Adaptive Evolution in Extreme Karst Environments
Source: Genomics Proteomics Bioinformatics. 2025 Feb 11;23(1):qzaf007. doi: 10.1093/gpbjnl/qzaf007 (PMC12231542; doi:10.1093/gpbjnl/qzaf007)
Supplement: qzaf007_Supplementary_Data [file FigS9.pdf › FigS9.pdf]

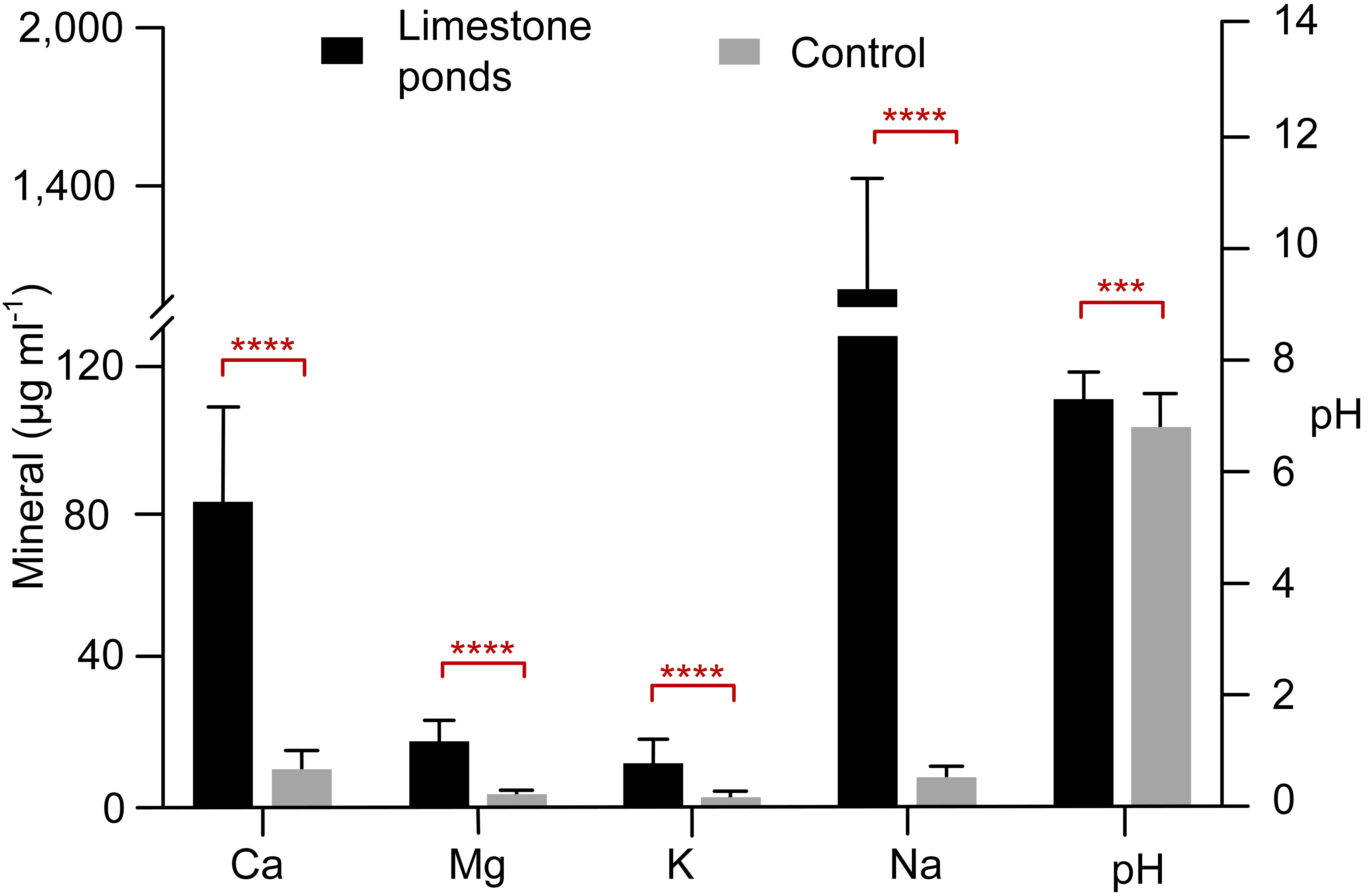

2,000
1,400
120
80
40
0
Limestone ponds
Control
****
****
****
****
***
Mineral (μg ml-1)
14
12
10
8
6
4
2
0
pH
Ca
Mg
K
Na
pH

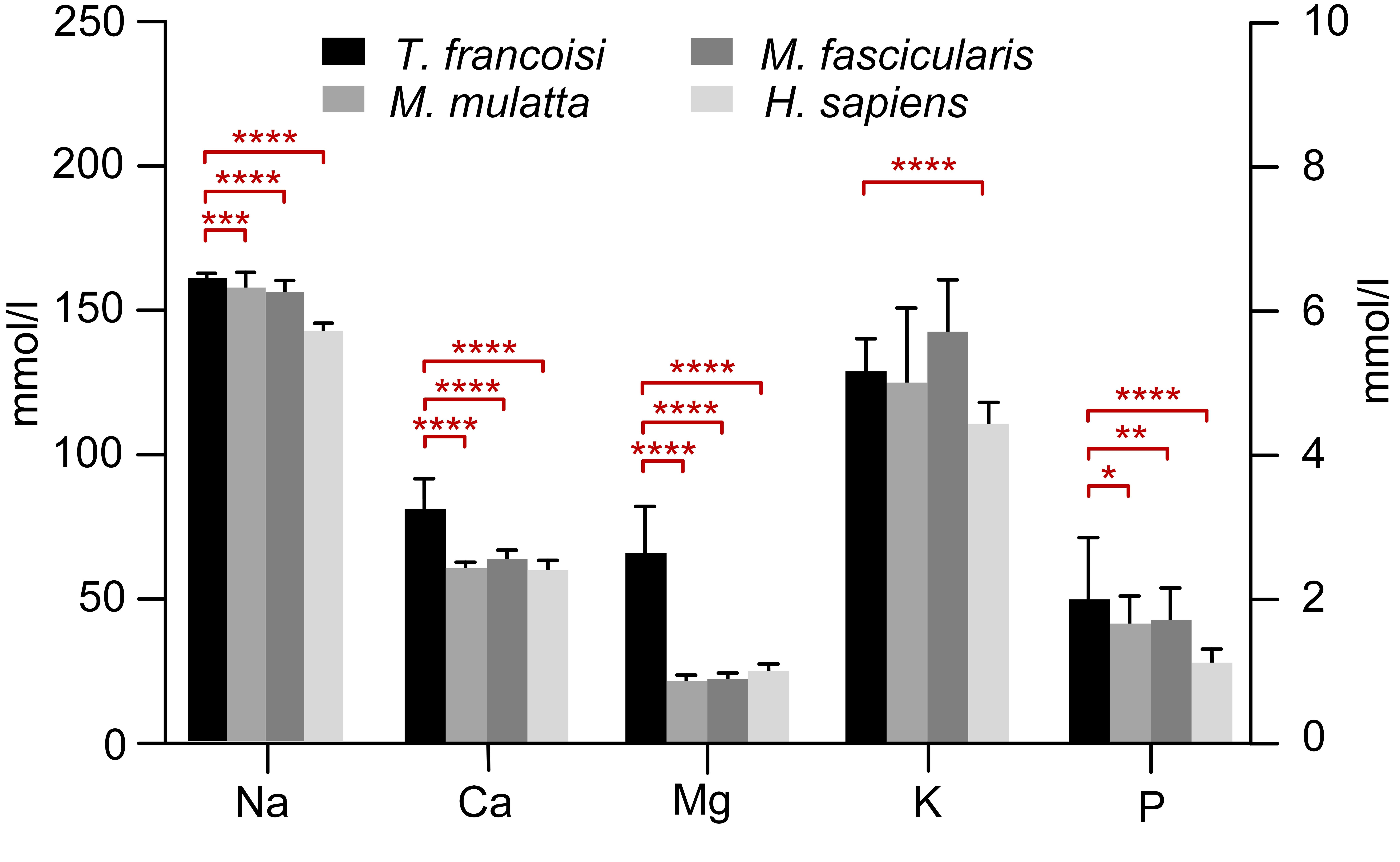

250
200
150
100
50
0
T. francoisi
M. mulatta
M. fascicularis
H. sapiens
mmol/l
10
8
6
4
2
0
mmol/l
Na
Ca
Mg
K
P
